# Supplementary material for: Long-term cognitive effects of menopausal hormone therapy: Findings from the KEEPS Continuation Study
Source: PLoS Med. 2024 Nov 21;21(11):e1004435. doi: 10.1371/journal.pmed.1004435 (PMC11581397; doi:10.1371/journal.pmed.1004435)
Supplement: S1 STROBE Checklist — (DOCX) [file pmed.1004435.s001.docx]

**Supplement**

**S1 Checklist**

STROBE Statement

|  | Item No | Recommendation | Page  No |
| --- | --- | --- | --- |
| **Title and abstract** | 1 | (*a*) Indicate the study’s design with a commonly used term in the title or the abstract | Abstract-First paragraph of the Methods and Finding section |
|  |  | (*b*) Provide in the abstract an informative and balanced summary of what was done and what was found | Abstract-Method and Finding section- first, second and third paragraph, Conclusion first paragraph. |
| Introduction | | | |
| Background/rationale | 2 | Explain the scientific background and rationale for the investigation being reported | Introduction section paragraphs: 2-3-4-5 |
| Objectives | 3 | State specific objectives, including any prespecified hypotheses | Introduction section paragraphs: 5 |
| Methods | | | |
| Study design | 4 | Present key elements of study design early in the paper | Abstract: Methods and Finding section, paragraph 1; Methods, subsection KEEPS Continuation participant, paragraph 1; Methods, subsection KEEPS and KEEPS Continuation, paragraph 1 |
| Setting | 5 | Describe the setting, locations, and relevant dates, including periods of recruitment, exposure, follow-up, and data collection | Abstract: Methods and Finding section, paragraph 1; Methods, subsection KEEPS Continuation participant, paragraph 1; subsection Enrolment sites and ethics approvals, paragraph; subsection KEEPS and KEEPS Continuation, paragraph 1; |
| Participants | 6 | (*a*) *Cohort study*—Give the eligibility criteria, and the sources and methods of selection of participants. Describe methods of follow-up  *Cross-sectional study*—Give the eligibility criteria, and the sources and methods of selection of participants | Methods, subsection KEEPS Continuation participant, paragraph 1; Methods, subsection KEEPS and KEEPS Continuation, paragraph 1. |
|  |  | (*b*) *Cohort study*—For matched studies, give matching criteria and number of exposed and unexposed |  |
| Variables | 7 | Clearly define all outcomes, exposures, predictors, potential confounders, and effect modifiers. Give diagnostic criteria, if applicable | Methods, subsection KEEPS and KEEPS Continuation, paragraph 1; Methods, subsection Primary outcomes, paragraph 1; Methods, subsection mHT use, paragraph 1; Statistical methods, subsection Primary analysis, Post hoc sensitivity analysis and Secondary analysis. |
| Data sources/ measurement | 8* | For each variable of interest, give sources of data and details of methods of assessment (measurement). Describe comparability of assessment methods if there is more than one group | *Methods subsection* KEEPS and KEEPS Continuation, paragraph 1; Statistical methods, subsection Primary analysis, Post hoc sensitivity analysis and Secondary analysis. |
| Bias | 9 | Describe any efforts to address potential sources of bias | *Methods subsection* Primary analysis paragraph 2; Discussion paragraph 9 |
| Study size | 10 | Explain how the study size was arrived at | Methods subsection KEEPS Continuation participants paragraph 1 |
| Quantitative variables | 11 | Explain how quantitative variables were handled in the analyses. If applicable, describe which groupings were chosen and why | Statistical methods paragraph 1; subjection Primary analysis, paragraph 1, 2; subsection Secondary analysis, paragraph 1 |
| Statistical methods | 12 | (*a*) Describe all statistical methods, including those used to control for confounding | Statistical methods, paragraph 1; subsection Primary analysis, paragraph 1; subsection Secondary analysis, paragraph 1 |
|  |  | (*b*) Describe any methods used to examine subgroups and interactions | Statistical methods, subsection Post hoc sensitivity analysis, paragraph 1; subsection Secondary analysis, paragraph 1 |
|  |  | (*c*) Explain how missing data were addressed | Statistical methods, subsection Primary analysis, second paragraph |
|  |  | (*d*) *Cohort study*—If applicable, explain how loss to follow-up was addressed  *Cross-sectional study*—If applicable, describe analytical methods taking account of sampling strategy | Statistical methods, paragraph 1; subsection Secondary analysis: Cross-sectional comparison, paragraph 1 |
|  |  | (*e*) Describe any sensitivity analyses | Statistical methods, subsection Post hoc sensitivity analysis, paragraph 1 |

Continued on next page

| Results | | | |
| --- | --- | --- | --- |
| Participants | 13* | (a) Report numbers of individuals at each stage of study—eg numbers potentially eligible, examined for eligibility, confirmed eligible, included in the study, completing follow-up, and analysed | Figure 2, Methods, subsection KEEPS Continuation participants, paragraph 1;subsection KEEPS and KEEPS Continuation, paragraph 1 |
|  |  | (b) Give reasons for non-participation at each stage | Figure 2, subsection KEEPS and KEEPS Continuation, paragraph 1 |
|  |  | (c) Consider use of a flow diagram | Figure 2 |
| Descriptive data | 14* | (a) Give characteristics of study participants (eg demographic, clinical, social) and information on exposures and potential confounders | Table 1 |
|  |  | (b) Indicate number of participants with missing data for each variable of interest | Table 1 |
|  |  | (c) *Cohort study*—Summarise follow-up time (eg, average and total amount) | Figure 1 |
| Outcome data | 15* | *Cohort study*—Report numbers of outcome events or summary measures over time | Table 1-2 |
|  |  |  |  |
|  |  | *Cross-sectional study—*Report numbers of outcome events or summary measures | *Table 3* |
| Main results | 16 | (*a*) Give unadjusted estimates and, if applicable, confounder-adjusted estimates and their precision (eg, 95% confidence interval). Make clear which confounders were adjusted for and why they were included | Table 1-3 |
|  |  | (b) Report category boundaries when continuous variables were categorized | Table 1-3 |
|  |  | (*c*) If relevant, consider translating estimates of relative risk into absolute risk for a meaningful time period |  |
| Other analyses | 17 | Report other analyses done—eg analyses of subgroups and interactions, and sensitivity analyses | Methods subsection Post hoc sensitivity analysis, paragraph 1 |
| Discussion | | | |
| Key results | 18 | Summarise key results with reference to study objectives | Discussion paragraph 1 |
| Limitations | 19 | Discuss limitations of the study, taking into account sources of potential bias or imprecision. Discuss both direction and magnitude of any potential bias | Discussion, paragraph 9. |
| Interpretation | 20 | Give a cautious overall interpretation of results considering objectives, limitations, multiplicity of analyses, results from similar studies, and other relevant evidence | Discussion section |
| Generalisability | 21 | Discuss the generalisability (external validity) of the study results | Discussion last three paragraph. |
| Other information | | | |
| Funding | 22 | Give the source of funding and the role of the funders for the present study and, if applicable, for the original study on which the present article is based | Funding |
