# Supplementary material for: Long-term cognitive effects of menopausal hormone therapy: Findings from the KEEPS Continuation Study
Source: PLoS Med. 2024 Nov 21;21(11):e1004435. doi: 10.1371/journal.pmed.1004435 (PMC11581397; doi:10.1371/journal.pmed.1004435)
Supplement: S1 Protocol — (DOCX) [file pmed.1004435.s002.docx]

**Prospective Observational Study**

**08 November 2017**

**FULL PROTOCOL TITLE**

Prevention of Alzheimer’s Disease in Women: Risks and Benefits of Hormone Therapy- Continuation of **“**The Kronos Early Estrogen Prevention Study (KEEPS)”

**Study Chairman or Principal Investigator:**

Kejal Kantarci, MD

Professor of Radiology

**Supported by:**

**The National Institute of Aging**

**1RF1 AG057547-01**

Table of Contents

[Study Team Roster 5](#_Toc497922376)

[Précis 6](#_Toc497922377)

[1. Study Objectives 7](#_Toc497922378)

[2. Background and Rationale 7](#_Toc497922379)

[2.1. Alzheimer’s disease represents a public health crisis, especially for women 7](#_Toc497922380)

[2.2. The KEEPS Continuation represents a singular opportunity 8](#_Toc497922381)

[2.3. Estrogens’ effects on neurobiology of AD – importance of the timong of exposure 9](#_Toc497922382)

[2.4. Seminal studies – WHIMS, WHISCA, KEEPS and ELITE 9](#_Toc497922383)

[2.5. Understanding the mechanisms of mHT effects on the brain through imaging biomarkers 10](#_Toc497922384)

[2.6. Overall Impact 11](#_Toc497922385)

[3. Study Design 11](#_Toc497922386)

[4. Selection and Enrollment of Participants 12](#_Toc497922387)

[4.1. Inclusion Criteria 12](#_Toc497922388)

[4.2. Exclusion Criteria 12](#_Toc497922389)

[4.3. Study Enrollment Procedures 12](#_Toc497922390)

[5. Study Interventions 12](#_Toc497922391)

[5.1. Interventions, Administration, and Duration 12](#_Toc497922392)

[5.2. Handling of Study Interventions 12](#_Toc497922393)

[5.3. Concomitant Interventions 13](#_Toc497922394)

[5.4. Adherence Assessment 13](#_Toc497922395)

[6. Study Procedures 13](#_Toc497922396)

[6.1. Schedule of Evaluations 13](#_Toc497922397)

[6.2. Description of Evaluations 14](#_Toc497922398)

[6.2.1. Screening Evaluation and Consent 14](#_Toc497922399)

[6.2.2. Enrollment, Baseline, and/or Randomization 14](#_Toc497922400)

[6.2.3. Follow-Up Visits 15](#_Toc497922401)

[7. Safety Assessments 16](#_Toc497922402)

[7.1. Specifications of Study Parameters 17](#_Toc497922403)

[7.2. Methods and Timing for Assessing, Recording, and Analyzing Safety Parameters 17](#_Toc497922404)

[7.3. Adverse Events and Serious Adverse Events 17](#_Toc497922405)

[7.4. Reporting Procedures 17](#_Toc497922406)

[7.5. Follow-up for Adverse Events 18](#_Toc497922407)

[7.6. Safety Monitoring 18](#_Toc497922408)

[8. Intervention Discontinuation 18](#_Toc497922409)

[9. Statistical Considerations 18](#_Toc497922410)

[9.1. General Design Issues 18](#_Toc497922411)

[9.2. Primary Objective 1 18](#_Toc497922412)

[9.3. Primary Objective 2 19](#_Toc497922413)

[9.4. Primary Objective 3 21](#_Toc497922414)

[10. Data Collection and Quality Assurance 21](#_Toc497922415)

[10.1. Data Collection Forms 21](#_Toc497922416)

[10.2. Data Management 22](#_Toc497922417)

[10.3. Quality Assurance 22](#_Toc497922418)

[10.4. MRI Image Analysis 22](#_Toc497922419)

[10.5. PET/CT Image Analysis 24](#_Toc497922420)

[10.6. Training 24](#_Toc497922421)

[10.7. Quality Control Committee 24](#_Toc497922422)

[10.8. Metrics 24](#_Toc497922423)

[10.9. Protocol Deviations 24](#_Toc497922424)

[10.10. Monitoring 24](#_Toc497922425)

[11. Participant Rights and Confidentiality 24](#_Toc497922426)

[11.1. Institutional Review Board (IRB) Review 24](#_Toc497922427)

[11.2. Informed Consent Forms 24](#_Toc497922428)

[11.3. Participant Confidentiality 25](#_Toc497922429)

[11.4. Study Discontinuation 25](#_Toc497922430)

[12. Committees 25](#_Toc497922431)

[12.1. Day-to-Day Management at Mayo Clinic 25](#_Toc497922432)

[12.2. External Advisory Committee 25](#_Toc497922433)

[13. Publication of Research Findings 26](#_Toc497922434)

[14. Supplements/Appendices 26](#_Toc497922435)

[15. References 26](#_Toc497922436)

[Appendix A: Laboratory Tests 33](#_Toc497922437)

[Appendix B: Brain MRI and PET/CT Acquisition and Post-Processing 34](#_Toc497922438)

[Appendix C: Pregnancy Questionnaire 35](#_Toc497922439)

# Study Team Roster

Kent R. Bailey, Ph.D.

Health Sciences Research

Biomedical Statistics and Informatics

(507) 284-5581

Assistant: Jen Voss (507) 284-5567

Kimberly J. Bailey Psychometrist

Mayo L-11

(507) 538-2135

N. Maritza Dowling, Ph.D.

George Washington University School of Nursing

[nmdowling@gwu.edu](mailto:nmdowling@gwu.edu)

Julie A. Fields, Ph.D., L.P.

Psychology and Psychiatry

Neurocognitive Disorders

(507) 293-4230

Carey E. Gleason, Ph.D.

University of Wisconsin, School of Medicine and Public Health,

William S. Middleton Memorial VA Hospital (11G) GRECC; 2500 Overlook Terrace

(608) 280-7000

[ceg@medicine.wisc.edu](mailto:ceg@medicine.wisc.edu)

Brieanna Harris

University of Wisconsin, School of Medicine and Public Health

K6/339, 3690 Clinical Science Center

600 Highland Ave

Madison, WI 53792

(608) 263-8620

[blharris@medicine.wisc.edu](mailto:blharris@medicine.wisc.edu)

Muthuvel Jayachandran, Ph.D.

Surgical Research

(507) 284-2481

Kejal Kantarci, M.D., M.S.

Neuroradiology

Center for Advanced Imaging Research

(507) 538-0762

[Kantarci.kejal@mayo.edu](mailto:Kantarci.kejal@mayo.edu)

Assistant: Heidi Morrow (507) 284-9770

Ekta Kapoor, M.D.

Women’s Health Research

General Internal Medicine

[Kapoor.ekta@mayo.edu](mailto:Kapoor.ekta@mayo.edu)

Timothy Lesnick

Health Science Research

Biomedical Statistics and Informatics

Harwick 7

(507) 266-0478

Val Lowe, M.D.

Radiology - Nuclear Medicine

(507) 284-9599

Assistant: Rose Busta (507) 284-4104

Virginia M. Miller, Ph.D.

Surgical Research

(507) 284-2290

Assistant: Marilyn Vail (507) 266-4011

Denise Reyes

Radiology

Center for Advanced Imaging Research

Opus 2 – 163

(507) 538-7263

[Reyes.denise@mayo.edu](mailto:Reyes.denise@mayo.edu)

Nirubol Tosakulwong

Health Science Research

Biomedical Statistics and Informatics

Harwick 7

Samantha Zuk

Radiology

Center for Advanced Imaging Research

Medical Imaging Analyst

# Précis

**Study Title**

Prevention of Alzheimer’s Disease in Women: Risks and Benefits of Hormone Therapy - Continuation of “The Kronos Early Estrogen Prevention Study (KEEPS)”

**Objectives**

The objective of this study is to evaluate the imaging biomarkers of Alzheimer’s disease and cognitive function, in women who participated in the Kronos Early Estrogen Prevention Study (KEEPS).

**Design, Outcomes, Interventions, and Duration**

Women (n=118) who enrolled in the KEEPS study at Mayo Clinic will be invited to participate.

The current investigation will include assessments conducted within a six-week interval over 2- 3 visits, consisting of blood tests, questionnaires, neurocognitive testing, and brain imaging.

**Interventions and Duration**

- Visit 1 (will take approximately 2.5 hours):
- Explanation of the study and confirmation of eligibility
- Measurement of height, weight, waist and hip circumference, blood pressure
- and pulse
- Review of medical history
- Blood tests
- Questionnaires about general health and well-being, quality of life, sleep,
- emotional health and mood and pregnancy history.
- Visits 2 and if needed 3 (will take approximately 5 hours total):
  - Cognitive tests
  - Blood pressure and heart rate
  - Brain magnetic resonance imaging (MRI)
  - Brain positron emission tomography (PET/CT)

Participants will be on study for up to 6 weeks while completing medical assessment, questionnaires, blood work, neurocognitive studies, brain MRI, brain PET/CT.

**Sample Size and Population**

Women (up to 118 total) in early menopause who were randomized to hormone therapy vs. placebo as participants in KEEPS will be invited to participate in this study.

# Study Objectives

To assess effects of menopausal hormone therapy and normal aging on cognitive performance and imaging markers of brain structure in women approximately twelve years after enrolling in the KEEPS study. KEEPS participants were randomized to oral or transdermal estrogen treatments or placebo within three years of menopause. This is a follow up study of these women approximately twelve years after randomization (8 years after study completion.) **No treatments are given as part of this study**; any current hormonal treatments are by choice and prescribed by the participant’s personal physician.

**Aim 1:** Determine the differences in Aβ, cerebrovascular lesions and brain structure in women who were treated with either oCEE or tE2 compared to placebo during early postmenopause.

*Hypothesis 1:* Aβ, cerebrovascular lesions and brain structure are different in women who were treated with tE2 vs. placebo and oCEE vs. placebo during early postmenopause, modified by the *APOE ε4* status.

**Aim 2:** Determine the differences in longitudinal changes in cognitive performance and mood in women who were treated with either oCEE or tE2 compared to placebo within three years of menopause.

*Hypothesis 2:* The longitudinal change in cognitive performance and mood over 12 years in women treated with one of the two active estrogen formulations (tE2 or oCEE) will differ from longitudinal cognitive and mood changes occurring in women who received placebo.

**Aim 3:** To determine the association between imaging markers investigated in Aim 1 and the change in cognitive performance investigated in Aim 2 seven to eight years after completing KEEPS trial participation.

*Hypothesis 3:* Higher Aβ, and WMH load, and smaller regional brain volumes are associated with a longitudinal decline in cognitive performance and mood outcomes since randomization.

# Background and Rationale

## Alzheimer’s disease represents a public health crisis, especially for women

Women represent approximately ⅔ of the five million patients with AD dementia. At the age of 65, a woman’s lifetime risk for AD is nearly double that for a man, 17.2% compared to 9.1%.[1] While it remains imperative to develop effective AD treatments, the prevailing sentiment is that initiating treatment *after* the onset of clinical symptoms may be “too little, too late” to alter disease course,[2, 3] necessitating a shift toward AD prevention in individuals who are at-risk. Thus, there is an urgent need for effective *prevention strategies* to address the suffering and untenable costs associated with AD. How menopausal hormone therapies (mHT) influence the risk of AD remains an area of controversy. As the number of postmenopausal women in the world is projected to be ~1.1 billion in less than a decade from today,[4] and that mHT is prescribed to relieve symptoms of menopause, it is critical that this controversy be addressed in rigorous manner. The results of the proposed research will address this controversy by applying principles of pharmacogenomics with state-of-the art imaging and cognitive testing in postmenopausal women whose hormonal usage and cardiovascular risk factors have been documented for over 10 years. The outcomes would provide evidence for a potential preventive strategy for women at risk.

## The KEEPS Continuation represents a singular opportunity

The KEEPS, and its ancillary KEEPS Cognitive and Affective Study (KEEPS-Cog) recruited women from 2005 to 2008; all participants were 6 to 36 months past their last menses (age=42-58). The goals of this randomized, double-blinded, placebo-controlled trial was to investigate the cardiovascular, cognitive, and mood effects of two forms of mHT (tE2 and oCEE) administered proximal to the menopausal transition to women whose cardiovascular risks were minimal.[5] In the Women’s Health Initiative (WHI) and its Memory Study (WHIMS), oCEE and medroxyprogesterone acetate initiated decades past the menopausal transition, increased the risk of dementia.[6] Since the WHI, prescribing practices have changed such that there are a wide variety of mHTs used to alleviate menopausal symptoms. Whether alternative formulations of mHT, such as tE2 used in KEEPS, can preserve neuronal integrity and decrease the risk of dementia when administered early in menopause remains controversial.[7-14] Our pilot data suggest possible differential effects for two mHT formulations. The inclusion of women who were at higher risk for cardiovascular disease and well past their menopausal transition were considered major design flaws of the WHI study[15] and WHIMS,[16, 17] respectively. The KEEPS and KEEPS-Cog were designed to address these limitations, and to determine whether lower doses and different formulations of mHT could reduce the risk of cognitive decline and mood changes that occur during menopausal transition, but only if initiated shortly after menopause during the “*critical window*”; that is, a time-point that would be consistent with a preventive strategy.[18] Women enrolled in the KEEPS are now ~12 years past their randomization to either oCEE or tE2 or placebo, and ~8 years past the termination of study treatment. They are an ideal cohort to investigate potential preventive aspects of mHT as their mHT use was carefully monitored during the clinical trial

Follow-up assessments in the KEEPS Continuation Study will occur at an age (median age=65), when Aβ abnormalities are most likely to dissociate healthy from early AD-related imaging biomarker changes.[19] ***Thus, participants of the KEEPS Continuation Study are at an ideal age to determine the long-term effects of two different formulations of mHT on preclinical AD pathophysiology*.** The consequences of Aβ deposition during early menopausal years are not fully understood, and effectiveness of early mHT for preventing AD-related pathology in the long-term remains unclear. However, reducing Aβ deposition through Aβ-modifying therapies is a widely accepted strategy for preventing AD, and clinical trials are underway in cognitively normal individuals with high Aβ deposition on PET,[2] and in *APOE ε4* carriers.[20] Therefore, the ability to leverage an identified cohort of women who are *APOE* genotyped, and were exposed soon after menopause to clinically relevant formulations of mHT represents an opportunity not to be missed. Moreover, we are well positioned to investigate the interplay of mHT, cerebrovascular disease, and longitudinal change in cognitive function. A recent examination of records from nearly 500,000 women found that estradiol mHT was associated with lower mortality from vascular dementia (VaD) and AD,[21] and the effect was pronounced for VaD, hinting at the underlying mechanisms of cognitive actions.[21]

As discussed in the Funding Opportunity Announcement (FOA), there is a great need to clarify *both* risk and protective exposures in midlife. ***Among the FOA research objectives, the KEEPS Continuation most clearly fulfills the proposal’s stated goal to ‘test whether putative risk or protective factor are truly causal.’*** Follow-up evaluation of this group of women, whose midlife mHT use and cardiovascular status were well-characterized, provides a singular opportunity to clarify the long-term effects of two formulations of mHT. For women considering mHT, the findings can provide critical insights, guiding their healthcare decisions.

## Estrogens’ effects on neurobiology of AD – importance of the timong of exposure

There is compelling evidence that estrogens influence development of AD neuropathology. Estrogens reduce inflammatory responses,[22, 23] especially to Aβ,[24] improve CSF clearance of insoluble Aβ,[25] while increasing expression of non-toxic soluble Aβ,[26] increase synaptogenesis and dendritic spine density in the hippocampal CA1 field,[27, 28] and prefrontal cortex,[29, 30] and exert antioxidant effects.[31] In addition, estrogens modulate metabolic function;[32] in particular, regulation of brain mitochondrial glucose transport and glycolysis.[33] However, estrogen’s effects may depend on the underlying health of the metabolic system. Brinton [34, 35] proposed the concept of a “healthy-cell bias,” which suggests that the health of the neuronal substrate will influence the effects of estrogen exposure. Specifically, estrogens increase mitochondrial respiration and ATP generation in healthy neurons, while protecting cells by improving tolerance for calcium influx and increasing antioxidant actions.[36-38] In aged or diseased cells, in which calcium homeostasis is disrupted, estrogen-induced calcium influx becomes deleterious to neurons.[34] Consistent with this supposition, Espeland et, al, recently reported that women with diabetes (age >65) randomized to oCEE in WHIMS demonstrated twice the risk for dementia,[39] and greater gray matter atrophy [40] compared to women without diabetes who were assigned to placebo. Importantly, non-diabetic women on oCEE did not show a greater risk for dementia than the reference group. Hence, the risks and benefits of mHT on cognitive function and AD pathophysiology may depend on a woman’s age, her overall health, and in particular, her metabolic and vascular health.[41]

## Seminal studies – WHIMS, WHISCA, KEEPS and ELITE

Two Women’s Health Initiative (WHI) ancillary studies, the WHI Memory Study (WHIMS) and WHI Study of Cognitive Aging (WHISCA) found that both opposed and unopposed oCEE were associated with adverse cognitive effects,[16, 42-45] and no mood benefits [44, 45] when initiated in women age 65 or older. Both treatment conditions were associated with greater brain atrophy than placebo.[46] To clarify the importance of the age at which mHT was initiated, i.e., proximity to menopause, WHI scientists examined the cognitive function of women enrolled in the WHI trial between the ages of 50 and 55 and found no evidence of cognitive benefit or harm more than a decade after mHT was initiated.[47] The KEEPS and its ancillary KEEPS-Cog, and the Early vs. Late Intervention with Estradiol (ELITE) trials were launched in order to address remaining controversies. In KEEPS, mHT was initiated close to the age of menopause; whereas ELITE compared women randomized to mHT within six years of menopause to women exposed more than 10 years past menopause.[32] A review of KEEPS and KEEPS-Cog is provided in our **Preliminary Studies**. Cardiovascular findings from ELITE suggested that early but not late intervention slowed atherosclerosis.[33] Interestingly, the ELITE cognitive trial found no difference in the cognitive effects of mHT based on timing of exposure.[48] Neither KEEPS-Cog nor ELITE has reported on ***long-term effects*** of mHT’s. In contrast to WHIMS findings, data from the Prospective Epidemiological Risk Factors (PERF) study suggest that younger women (mean age 54.1) randomized to mHT performed better on cognitive outcomes than women treated with placebo, 5 to 15 years after study involvement ended.[49] It should be noted that in addition to differences in timing of mHT, the formulations of hormones differed among studies. In the WHI, the formulations were oCEE with medroxyprogesterone acetate, a synthetic progestogen.[50] In the ELITE trial, oral 17β-estradiol plus micronized progesterone was used.[32] In the KEEPS, two formulations were compared to placebo: oCEE at a lower dose than in the WHI was used (0.45mg/day) and transdermal 17β-estradiol; each paired with a pulsed micronized progesterone.[5] The formulations used in KEEPS are those most commonly used in clinical practice today. Thus, the KEEPS Continuation study will address inconsistencies in findings, clarifying the effects of mHT on AD biomarkers and cerebrovascular contributions and provide the needed information regarding the long -term cognitive effects of mHT treatments used in current clinical practice.

## Understanding the mechanisms of mHT effects on the brain through imaging biomarkers

F-18 Florbetapir PET/CT directly measures the β-amyloid (Aβ) pathology of AD.[51] A positive Aβ PET scan is proposed as a research criterion for preclinical AD.[52, 53] Carriers of the *APOE* ε4 allele are at an increased risk of AD dementia; moreover the risk may be higher in women than in men. [54-56] *APOE ε4* carriers have increased Aβ deposition at an earlier age than *APOE ε4* non-carriers, and this difference is more pronounced in women than in men. [57, 58] Thus, women who are *APOE ε4* carriers are at a higher risk for AD-related Aβ deposition and may benefit most from early initiation of preventive interventions. The age of KEEPS participants we plan to recruit for the current project will be between ages 54 to 70 (median age=65). In the population-based Mayo Clinic Study of Aging (MCSA), 18% of the women have a positive Aβ PET scan at this age range. Aβ PET scan positivity is approximately three times higher in women who are *APOE ε4* carriers (33% positive) compared to *APOE ε4* non-carriers (11% positive) in this age range. Similarly, in the Longitudinal Baltimore Study of Aging, *APOE ε4* positivity conferred a three-fold risk of accumulating Aβ after adjusting for sex and education.[59] Although estrogens are thought to modify AD risk, there are only limited data on the estrogen effects on Aβ.[60]

Effects of mHT on brain morphology have been investigated in cross-sectional observational MRI studies, with varying findings in cognitively normal postmenopausal estrogen users compared to non-users.[21, 61-66] As for all observational studies, these imaging studies are subject to “healthy user bias”. Contrary to the findings from observational studies, data from WHIMS indicate greater hippocampal atrophy in postmenopausal women who are treated with oCEE at age 65 years and older.[46] In WHIMS, women with low baseline cognitive function and high ischemic WMH burden were more prone to this treatment effect on the hippocampus, suggesting greater vulnerability to mHT-associated atrophy for already compromised brains.[46, 67] Furthermore, hippocampal volumes correlated with cognitive function in the treated group, suggesting oCEE induces cognitive impairment through increased brain atrophy.[68] WMHs were associated with baseline blood pressure in WHIMS, and a greater longitudinal increase in WMH occurred in those with higher blood pressure demonstrating the longitudinal blood pressure effects on the ischemic WMH.[69] MRI findings in WHIMS are consistent with the previously reported decline in cognitive function and increased risk of dementia with oCEE in this cohort, and demonstrate that MRI-based measures of brain morphology are robust biomarkers of cognitive function in postmenopausal women. Additionally, there is evidence that WMH load is associated with small vessel disease in the brain.[67, 70] Hypertensive renal disease is strongly associated with WMH,[71] and a better control of blood pressure slows WMH progression.[72, 73] An association between WMH load and future risk for mild cognitive impairment is established.[74-76] The KEEPS Continuation Study will examine these underlying disease-associated changes, relating to long -term cognitive effects of two formulations of mHT.

## Overall Impact

Findings from the KEEPS Continuation study hold the potential to alter the clinical practice paradigms related to treatment of menopausal symptoms and prevention of AD. For the more than 4.5 million postmenopausal women in the world and the women currently using mHT, clarifying the long-term effects of different types and formulations of mHT on the brain is critical.

The KEEPS Continuation study will be unique in clarifying inconsistencies in the literature regarding mHT and cognitive health in the following ways: 1) Provide information regarding potential preventive or risk effects of clinically relevant formulations of mHT on AD biomarkers and cerebrovascular lesions; 2) Provide 12 year follow-up of cognitive change in women who initiated mHT early in menopause; 3) Evaluate the efficacy of mHT relative to *APOE ε4*.

# Study Design

This is a continuation of the Kronos Early Estrogen Prevention Study (KEEPS) (KEEPS; NCT00154180; Mayo Clinic IRB #2241-04-00), a multi-center double blinded, placebo-controlled, randomized trial (NCT00154180) funded by the Kronos Longevity Research Institute, Phoenix, AZ to test the hypothesis that hormone therapy started early in menopause (within the **"window of opportunity**”) would slow progression of atherosclerosis as measured by changes in carotid artery intima-medial thickening and coronary arterial calcification**.** Enrollment in the KEEPS ended in 2008 with the final study visits occurring in late 2012.[77] Primary and secondary outcomes of the KEEPS targeted progression of atherosclerosis, measured as changes in carotid artery intima–media thickness (CIMT) by ultrasonography and coronary artery calcium (CAC) score. Both CIMT and CAC quantify atherosclerosis changes; predict risk for cardiovascular events; and are altered with mHT.[78, 79] 727 healthy menopausal women aged 42 to 58 years at entry, all within 36 months from last menses without prior CVD events were enrolled at nine US academic sites. All women had a coronary artery calcium (CAC) score < 50 Agatston units and had not received estrogen or lipid-lowering therapy for > 3 months. Women were randomized to 48 months of placebo or mHTs with either 0.45 mg/d oCEE, or 50 mcg/d tE2, and with each mHT, 200 mg of oral progesterone was administered for the first 12 days of each month. After 4 years of mHT there were no differences between treatment groups and mean CIMT or CAC score increases. Contrary to hypotheses, four years of mHT did not alter progression of atherosclerosis in these generally healthy early postmenopausal women.

# Selection and Enrollment of Participants

This proposal is developed as a continuation of the Kronos Early Estrogen Prevention Study (KEEPS; NCT00154180; Mayo Clinic IRB #2241-04-00). At Mayo Clinic, 118 women met the inclusion criteria for randomization into the KEEPS study. This study will recruit these 118 menopausal women for follow-up. Assuming a 70% retention rate, we project that our cohort will consist of approximately 82 subjects.

## Inclusion Criteria

Participants must meet all of the following inclusion criteria in order to participate in this study:

- Enrolled into the KEEPS study and randomized to one of the treatment or placebo arms,
- able to understand study procedures, and
- willing to sign an authorization of consent in order to participate in this study.

## Exclusion Criteria

Women who have contraindications to MRI for safety reasons, such as an MRI-incompatible implant or claustrophobia will be excluded only from the imaging studies (MRI and PET/CT).

## Study Enrollment Procedures

Once IRB approval has been obtained, a standard IRB approved recruitment and informed consent process will be followed. A sample of 118 women who participated in the KEEPS study at Mayo Clinic who meet the inclusion criteria will be considered eligible for the study. Investigators will provide names and contact information for all 118 women who previously participated in the KEEPS study. Participant names and contact information will be recorded directly into the Medidata Rave database (described below) on a secure institutional server at the Mayo Clinic. This server will be accessible only to study staff. A study coordinator will send eligible participants a letter by mail and invite them to participate.

Eligible participants will be asked to call the study coordinator if interested, or to mail a response card indicating no interest in further contact. For interested individuals, the study coordinator will provide a detailed description of what the study involves and will collect information on current use of medication and interim cardiovascular, cerebrovascular, or neurologic disease events. Individuals who meet eligibility criteria will scheduled for a study visit at the Mayo Clinic Clinical Research Unit (CRU) of the Center for Translational Science Activities. Participants will be instructed to fast for 12 hours prior to that visit.

# Study Interventions

## Interventions, Administration, and Duration

None; this is a follow-up study.

## Handling of Study Interventions

Not applicable

## Concomitant Interventions

None

## Adherence Assessment

Not applicable

# Study Procedures

## Schedule of Evaluations

Study participants will be scheduled to complete the following assessments and tests within a six week interval (42 days) over 2-3 visits:

**Medical history** will include reproductive history (including pregnancy-related disorders such as gestational diabetes or hypertensive disorders), cardiovascular and cerebrovascular symptoms or conditions, and use of medications including mHTs (about 30 min).

**Clinical examination** will include blood pressure, pulse, height, weight, and waist-hip measurement and examination for the diagnosis of MCI according to the National Institute on Aging-Alzheimer's Association (NIA-AA) criteria,[80] or dementia according to DSM V (about 30 min).

**Blood will be collected** for testing of the lipids, fasting glucose, Vitamin B12 and TSH levels, C-reactive protein, hemoglobin A1c, DNA and PaxGene RNA. Kits will be shipped to the sites and sites will ship the samples back to Mayo Clinic in batches (about 30 min).

Fasting: Only subjects who can safely fast for 12 hours will be asked to provide fasting blood samples. Included in the examination are laboratory tests requiring a collection of approximately 40 mLs or 8 Tsp. or 2.6 Tbsps. of blood after a 12 hour fast. Subjects will be provided a light breakfast immediately after this procedure. Participants will have the fasting procedure and risks explained to them and give verbal consent to fast via the screening interview prior to their baseline visit. The screening interview will identify participants who are diabetic and/or have questions or concerns with fasting. Those identified as having questions or concerns will have a phone call scheduled with a clinician to discuss safe fasting procedures prior to the baseline visit. If, after this phone call, a participant indicates that (s)he has further concerns, (s)he will be instructed that (s)he may call an ADRC clinician to discuss any other questions on fasting. A fasting tip sheet will also be mailed to participants with their visit reminder letter. The tip sheet explains the risks of fasting, instructions for ending the fast, and a number to call if they have further questions.

Blood samples: The specific laboratory tests for which blood will be collected are listed in [**Appendix A**](#_Appendix_A:_Laboratory). In addition to the blood collected for laboratory tests, some blood will be used to derive serum and plasma (approximately 10 mL of each) samples to be stored for future use.

**Questionnaires,** assessing menopausal symptoms (Menopause Rating Scale - MRS), and quality-of-life (Utian Quality of Life Scale – UQOL) will be administered. Sleep quality will be assessed using the Pittsburg Sleep Quality Index (PSQI) Profile of Mood States (POMS), the Beck Depression and Anxiety Inventories (BDI and BAI) and the Baseline Sleep Quality Index Memory Function Questionnaire, Brief Patient Health Questionnaire. Data will be electronically transferred to UW (about 1 hour).

**Cognitive testing**, including Modified Mini-Mental State Exam, California Verbal Learning Test-II, New York University paragraph recall tests, Benton Visual Retention Test, Subtests from the Wechsler Memory Scale-III (Digit Span and Letter Number Sequencing subtests), Stroop Color Word Test, Trail Making Test A & B, Digit Symbol Coding from the Wechsler Adult Intelligence Scale – 3, Letter Fluency and Category Fluency tests. Data will be electronically transferred to UW (about 2 hours).

**Brain** MRI (about 1 hour).

**Brain** PET/CT (about 4 hours).

| **Table 1. Schedule of Evaluations** | | |
| --- | --- | --- |
|  | **Visit 1** | **Visits 2 and 3** |
| Informed consent | X |  |
| Medical history | X |  |
| Clinical exam | X |  |
| Surveys | X |  |
| Blood sample | X |  |
| Cognitive testing |  | X |
| Brain MRI |  | X |
| Brain PET/CT |  | X |

## Description of Evaluations

## Screening Evaluation and Consent

Informed consent: Women meeting eligibility criteria by phone screen will be scheduled to report to the Clinical Research Unit (CRU) located at the Charlton Building. The study coordinator will obtain informed consent.

## Enrollment, Baseline, and/or Randomization

Initial enrollment visit: After obtaining informed consent, a basic health assessment will be obtained through interview by the study coordinator, including cardiovascular and cerebrovascular symptoms or conditions, menopausal symptoms, and use of medications. Physical measurements will be obtained by CRU staff, including blood pressure, pulse, height, weight, and waist-hip circumference, and a fasting venous blood sample (about 5 tablespoons) will be collected for analysis of lipid panel, fasting glucose, Vitamin B12 and TSH levels, C-reactive protein, hemoglobin A1c, and DNA (See [Appendix A](#_Appendix_A:_Laboratory) for listing of laboratory tests). Questionnaires will be administered. Appointments will be scheduled for the remainder of testing to be conducted over 2-3 follow-up visits. The entire visit will take approximately 2 hours.

Baseline assessments: This is a follow-up study; single tests will be performed.

Randomization: Not applicable

## Follow-Up Visits

**Visits 2 and 3** will be conducted within a two-week interval, to include cognitive testing, brain MRI and brain PET/CT.

Cognitive testing: A comprehensive battery of standardized neuropsychological tests will be administered by an individual trained by personnel overseeing the cognitive aim (Primary Objective 2) from the University of Wisconsin, Madison, (PI, Dr. Carey Gleason). At the Mayo Clinic site, neuropsychological tests are administered in the Research Psychometrics Resource at Mayo Clinic's Center for Translational Science Activities (CTSA) under the direction of Dr. Julie Fields.

The battery will consist of tests used in the original KEEPS study. [81] In addition to one global measure (Modified Mini-Mental test), all tests have been shown to load on one of four factors derived from data reduction procedures described previously.[82]

The four domains of cognitive performance will include:

1. Verbal Learning & Memory function using a composite domain z-score from California Verbal Learning Test (CVLT) and New York University (NYU) Paragraph tests
2. Auditory Attention & Working Memory function using a composite domain z-score from Wechsler Memory Scale-III Letter-Number Sequencing and Digit Span subtests
3. Visual Attention & Perceptual Speed using a composite domain z-score from Benton Visual Retention Test, Trail Making Test parts A and B, Stroop, and Digit Symbol Coding tests
4. Speeded Language & Mental Flexibility using a composite domain z-score from Letter (FAS) Fluency and Category (Animals, Fruits, Vegetables) Fluency tests

Brain MRI: This test will be obtained at the Radiology Department imaging facilities at the Charlton North Building. All MRI studies will be performed on a single 3T system (MAGNETOM, Siemens). A second 3T system with similar hardware and software will be identified as a back-up scanner. All MRI sequences used for this study will be acquired in a single sitting with an exam time under 45 min. The operating conditions of the scanner are controlled by its software, and will be in strict adherence to the non-significant risk guidelines as determined by the IRB and as defined by the FDA. The entire system is operated by commercially-available, 510(k) cleared software.

Brain PET/CT imaging: This test will be obtained at the Radiology Department imaging facilities at the Charlton North Building. After a 25-minute uptake period, the patient will be positioned on the scanner bed with instructions to remain motionless. Each participant will be injected with 10 mCi of F-18 Florbetapir (target dose 370 MBq, range 296 - 444 MBq). After a 25-minute uptake period, the patient will be positioned on the scanner bed with instructions to remain motionless. A helical CT image will be obtained at 50 minutes after injection of F-18 Florbetapir, followed by a 10-minute PET acquisition consisting of two 5-minute dynamic frames.

# Safety Assessments

Participation in this study may involve some discomforts or risks:

Blood draws: The problems associated with blood drawing include discomfort from

insertion of the needle (common), fainting at or about the time of blood drawing (infrequent), bruising at the site of the blood drawing (infrequent), and a clot or infection at the same site (rare).

Questionnaires: Participants may experience emotional response to questions. The Beck Depression Inventory asks about suicidal ideation. If a participant indicates that she has thoughts of suicide, a clinician will be paged to conduct a safety evaluation.

1. *Suicidality*

Psychometricians administering questionnaires will review the PDSQ before the patient leaves the appointment. If a participant endorses items on the PDSQ or states in context of other interviews that (s)he is suicidal (e.g., GDS), the psychometrician will page a study clinician. A study clinician, either a physician or a licensed psychologist, will meet with the study participant and conduct a suicide risk assessment. The clinician will use the following Columbia-Suicide Severity Rating Scale (C-SSRS) screening questions to conduct the assessment to ensure consistency among clinicians:

1. Have you wished you were dead or wished you could go to sleep and not wake up?
2. Have you actually had any thought of killing yourself?
3. (If no to both 1 & 2, skip to #6)
4. Have you been thinking about how you might kill yourself?
5. Have you had these thoughts and had some intention of acting on them?
6. Have you started to work out or worked out the details of how to kill yourself? And do you intend to carry out this plan?
7. Have you done anything, started to do anything, or prepared to do anything to end your life?

(If yes to 4, 5, &/or 6, take emergent action. Initiate 1:1 observation, complete full C-SSRS or obtain emergent health consultation).

Referral and recommendations will be made as needed. In cases where alcoholism is found in the subject’s parent (who will not be present), there will be no plans for referral.

1. *Depression*

Psychometricians administering questionnaires will review PDSQ and GDS before the patient leaves the appointment. If a participant endorses items or states in context of other interviews that the participant is depressed, the psychometrician will page a study clinician (usually a nurse practitioner). The clinician will meet with the study participant to discuss referral recommendations for clinical care (e.g., available psychologists in UW Health network). If the nurse practitioner feels immediate care is needed, they will page a licensed psychologist or physician to talk with them further to determine how to minimize further risks to subjects before an appointment with a psychologist can be made.

Cognitive testing: It is possible that anxiety may result from the neuropsychological testing.

Brain MRI: Individuals with claustrophobia may feel too confined and may not tolerate MRI scanning. If this occurs, the MRI scan will be stopped. Individuals will wear earplugs during the scan to reduce the discomfort from noise by the MRI machine. See [Section 7](#_Safety_Assessments) for information about MR imaging risk and safety.

Brain PET/CT: During PET/CT imaging, participants will be exposed to radiation from x-rays and radioactive materials. The amount of radiation exposure has a low risk of harmful effects.

*PET compound*: The radioactive mixture will be injected in the study participant’s vein (intravenous). This can result in a risk of pain or bruising or infection at the site of the needle stick. F-18 Florbetapir is an agent with specific high affinity for aggregated amyloid similar to other Thioflavin-T analogs and is non-toxic. This compound is cleared from the body within minutes and no toxic effects have been recorded with doses used in the study. As with any medication, allergic reactions are a possibility.

All participants will receive full supportive care while participating in the protocol. Mayo Clinic physicians remain on-call 24 hours per day to respond to any questions regarding subjects health concerns while participating in this protocol.

Participants may choose to discontinue participation in the study at any time

## Specifications of Study Parameters

Unexpected abnormal results on any testing completed as part of participation in this study will be reviewed by the Principal Investigator team and reported to the participant in a timely manner depending on the urgency and clinical significance of the abnormality.

## Methods and Timing for Assessing, Recording, and Analyzing Safety Parameters

The Principal Investigator or designee will review all reported side effects up to 8 weeks after the patient’s study to assess adverse events. Any adverse event reported to either the Principal Investigator or his designated research associates by the subject or medical staff caring for the subject will be recorded and the nature and attribution of cause of the event will be discussed. Any new adverse event that is also attributable to the study will be documented as such.

## Adverse Events and Serious Adverse Events

An adverse event includes both an expected side effect that is of a serious nature or an unexpected side effect/event regardless of severity. Any new, serious adverse events as described by Common Terminology Criteria for Adverse Events (CTCAE) v3.0 as found at [http://ctep.cancer.gov/for](http://ctep.info.nih.gov/CTC3/ctc_ind_term.htm))[ms/CTCAEv3.pdf](http://ctep.info.nih.gov/CTC3/ctc_ind_term.htm)) that are described will be reported to the IRB within 24 hours and the trial will be suspended immediately for review by the IRB. No serious side effects from the research imaging methods in this protocol are conceivable or expected.

## Reporting Procedures

Any adverse event that is so determined, or reported otherwise to either the Principal Investigators or their designated research associates by the subject or medical staff caring for the subject and which meets the criteria for a new adverse event that is also attributable to the study will be documented as such.

## Follow-up for Adverse Events

The Principal Investigator or designee will review all reported side effects up to 8 weeks after the patient’s study (reviews done on a monthly basis) to assess adverse events.

## Safety Monitoring

As noted in [Section 7.2](#_Methods_and_Timing).

# Intervention Discontinuation

Attributable adverse events will be reported to the IRB and the study suspended for discussion when needed.

# Statistical Considerations

## General Design Issues

The proposed analytical plan will comprise 1) data cleaning and basic statistical analyses to identify potential outliers, assess for normality, and examine variation in each variable; 2) the generation of standard descriptive statistics summarizing the sample and its characteristics; and 3) statistical methods for analyzing each specific aim and hypothesis. For each aim, all calculated *P* values will be 2-sided and *P*<0.05 will be considered statistically significant.

## Primary Objective 1

**To determine the differences in Aβ, cerebrovascular lesion load and brain structure in postmenopausal women who were treated with one of two mHTs vs. placebo after 12 years post-randomization and 8 years after the end of mHT administration phase.**

**Primary Hypothesis:** Aβ PET SUVR (primary outcome), WMH volume and regional cortical thickness (or hippocampal volume) are different in women who were treated with tE2 vs. placebo and oCEE vs. placebo.

**Hypothesis 1a:** Aβ PET SUVR (primary outcome), WMH volume and regional cortical thickness (or hippocampal volume) differences in women who were treated with tE2 vs. placebo and oCEE vs. placebo are modified by the *APOE ε4* status (carrier/non-carrier).

**Hypothesis 1b:** Aβ PET SUVR (primary outcome), WMH volume and regional cortical thickness (or hippocampal volume) differences in women who were treated with tE2 vs. placebo and oCEE vs. placebo during early postmenopause are modified by the vascular disease risk at 12 years post-randomization.

**Statistical Analysis for Aim 1:** We will first summarize the data for Aim 1 using standard descriptive statistics and a variety of plots (histograms, scatterplots) to assess variable distributions. Our outcome measures: Aβ PET SUVR (primary outcome), WMH volume, and regional cortical thickness (or hippocampal volume) for this aim will all be continuous cross-sectional variables, we will use analysis of covariance (ANCOVA) to address the primary hypothesis. Treatment groups (tE2, oCEE, and placebo) will be included as the predictors of interest, coded so that placebo is the reference group. Standard methods (Least Significant Difference, Tukey’s Honest Significant Difference, and Scheffé’s test) will be used to assess pair-wise differences. We will include a set of dummy variables to test and adjust for site (block) effects. The outcome variables will be transformed as necessary to meet regression assumptions underlying the ANCOVAs. These analyses will include baseline age, time from baseline, and *APOE ε4* status (as appropriate) as covariates. We will test for interactions of treatment with *APOE ε4* status through inclusion of two-way interactions in the models, and analyses in separate *APOE ε4* non-carrier and *APOE ε4* carrier strata. To address Hypothesis 1a, we will expand the set of covariates to include a vascular disease risk score assessed at the time of mHT/placebo initiation [82] or individual cardiovascular risk factors (systolic and diastolic BP, fasting glucose, smoking history, lipid profile, BMI, waist circumference). Possible interactions with these covariates will be handled in the same way as *APOE ε4* status. Hypothesis 1b will be treated much the same way, but CVD risk scores will be constructed from data acquired at 12 years after initiation of mHT. Since some of the women in the study might have used mHT after the trial ended, we will perform a series of sensitivity analyses with and without those individuals to assess any important effects and ensure that the final conclusions are robust.

**Power for Aim 1:** Focusing on F-18 Florbetapir PET/CT, our primary outcome variable for Aim 1, we first estimated the standard deviations in three groups of women (all subjects, *APOE ε4* non-carriers, and *APOE ε4* carriers) from the Mayo Clinic Study of Aging, a longitudinal study on aging and dementia.[83] We identified women with an age range of 55-70 (mean age=65) similar to our projected sample, who underwent both Aβ PET imaging and *APOE ε4* testing (n=264) Using these standard deviations as estimates of variability in our projected sample, we estimated the minimum detectable effect sizes in the pair-wise comparisons (using Cohen’s d) with 80% power.

| **Table 2. Minimum detectable effect sizes for projected sample size and 80% power.** | | | |
| --- | --- | --- | --- |
| **Comparisons** | **All** | ***APOE ε4*** **non-carriers** | ***APOE ε4* carriers** |
| oCEE vs. Placebo | 0.308 | 0.358 | 0.619 |
| tE2 vs. Placebo | 0.312 | 0.355 | 0.665 |

These effect sizes fall in the medium (all and *APOE ε4* non-carriers) to large (*APOE ε4* carriers) range. In our 84 month KEEPS data, most of our observed Cohen’s d values were close to 0.34 (all subjects), 0.37 (*APOE ε4* non-carriers), and 1.18 (*APOE ε4* carriers). We anticipate the effect sizes to be at least this large in the KEEPS Continuation study. Since the minimum detectable effect sizes in **Table 3** are close to or smaller than these values, we anticipate having adequate power to detect pairwise differences. We would expect similar effect sizes for the other outcomes.

## Primary Objective 2

**To determine the differences in longitudinal changes in cognitive performance and mood in postmenopausal women who were treated with one of the two mHTs vs. placebo.**

**Primary Hypothesis 2:** Performance on four cognitive factors described in our bi-factor model and on POMS mood indices are different in women who were treated with tE2 vs. placebo and oCEE vs. placebo.

**Hypothesis 2a:** Cognitive and mood differences in women who were treated with tE2 vs. placebo and oCEE vs. placebo are modified by *APOE ε4* status (carrier/non-carrier).

**Hypothesis 2b:** Cognitive and mood differences in women who were treated with tE2 vs. placebo and oCEE vs. placebo are modified by the vascular disease risk at 12 years post-randomization.

**Statistical Analysis for Objective 2:** Analyses for this aim will focus on longitudinal cognitive outcome data collected over six predetermined and unequally-spaced time points over a total period of 12 years. We will employ linear mixed-effects (LME) regression models with treatment groups (oCEE and tE2) vs. placebo (and their interactions with time) as predictors of cognitive change, and baseline age, *APOE ε4,* and education as control variables. Separate models will be estimated for each of the cognitive factor scores and mood outcome measures. The cognitive factor scores include the following four domains: verbal learning and memory; auditory attention and working memory; visual attention and perceptual speed; speeded language and flexibility. The mood outcome will be measured with the Profile of Mood States (POMS). The LME model incorporates covariance structures to account for the correlation between repeated measures across time. The analyses for the LME model will be conducted using the limited information maximum likelihood complete sample approach to missing data. The incorporation of higher order terms in the mixed-effect models will also be examined. All models will test and adjust for site effects. The shape of the trajectories across time will be inspected by plots and models will account for possible non-linearity. Random effects will be evaluated by likelihood-ratio (χ2) tests and fixed-effects will be evaluated via F-tests based on Type III sums of squares. Quantile-quantile plots of residuals will also be examined for evidence of significant outliers. As in Aim 1, we will address Hypothesis 2a by adding to the models a vascular disease risk score assessed at the time of mHT/placebo initiation (84) or individual cardiovascular risk factors. Possible interactions with these covariates will be handled in the same way as *APOE ε4* status. Similarly, for Hypothesis 2b, we will derive CVD risk scores from data acquired at 12 years after initiation of mHT. Since some of the women in the study might have used mHT after the trial ended, we will perform a series of sensitivity analyses with and without those individuals to assess any important effects and ensure that the final conclusions are robust.

**Power for Objective 2:** Power and sample size estimates to detect significant changes in cognitive function are based on Mayo Clinic pilot data collected over five unequally-spaced observation time points (0,18, 36, 48, and 84 months) with the verbal learning and memory factor as the longitudinal outcome and treatment groups vs. placebo as predictors. We assumed normally distributed continuous outcomes with missing data, two predictors (oCEE and tE2) with regression coefficients in factor score units for rate of change of, respectively, -0.20 and -0.11; a variance of 2.09 for the random intercept, a variance of 0.03 for the random slope, and a correlation between random slope and random intercept terms set to 0.07. Using a two-tailed *α*=0.05, a total sample size of 482 individuals will achieve a power of 82% to reject the hypothesis for this aim.

**Alternative Strategy:** We will evaluate our participants for incident MCI and dementia, but we do not expect to have sufficient power to test the differential progression to MCI or dementia in each of the mHT vs. placebo groups. However, we will explore this alternative approach if we have a sufficient number of incident cases.

## Primary Objective 3

**To determine the associations between imaging biomarkers investigated in Aim 1, and longitudinal changes in cognitive performance and mood investigated in Aim 2.**

**Primary Hypothesis 3:** Higher Aβ, and WMH load, and smaller regional cortical thickness are associated with a longitudinal decline in cognitive performance since randomization.

**Statistical Analysis for Objective 3:** Repeated cognition and mood measures will be available for time points ranging from trial baseline to 12 years post-baseline; we will use the same cognitive factor scores and POMS as in Aim 2. For each measure in Aim 2, we will consider the values at year 12, the changes in values from baseline to year 12, and predicted annual changes in values at year 12 (slopes). Aβ PET SUVR, WMH, and AD-signature thickness and hippocampal volume will only be available at 12 years post-baseline. We will use plots and Pearson or Spearman correlation coefficients, as appropriate given the distributions of the variables involved, to describe and measure associations between cognitive/mood measures and imaging biomarkers. As in the previous aims, we will explore and include site and covariate effects through regression models, transform variables as needed to meet assumptions, and perform sensitivity analyses with and without individuals who continued to use mHT after the mHT administration phase ended.

**Power for Objective 3:** With 482 individuals and α=0.05, we will have 80% power to detect Pearson correlation coefficients as small as 0.127. In a linear regression, this would be an increase in the model R^2^ due to the association of interest as small as 0.016. We should thus have sufficient power to detect small associations.

# Data Collection and Quality Assurance

## Data Collection Forms

Case report forms (CRFs) have been developed in paper format and will also be made available as electronic CRF (eCRFs), or data entry screens, using infrastructure from the clinical trial management system Medidata Rave. Participant source data will be entered directly or transcribed into the eCRFs or captured on paper forms first and then transcribed into the corresponding eCRFs. In particular, source data obtained at Visit 1, including medical history, current use of medications, vital signs and questionnaires will be captured on paper forms and entered in eCRFs.

Paper CRF for cognitive and mood data will be stored at sites where data are collected. Study coordinators will enter coded data through a password protected, secure, web-based data portal Medidata Rave. All cognitive and mood data will be stored on a HIPAA-compliant, secure University of Wisconsin server. Keys linking identity of participants to coded data will remain at the data collection sites.

MR images are stored in the Radiology clinical archiving system, and will be transferred to Mayo Clinic designated secure servers after anonymization. PET/CT images are stored in the Nuclear Medicine archiving system and will be transferred to Mayo Clinic designated secure servers after anonymization.

## Data Management

Participant source data will be entered directly or transcribed into eCRFs using Medidata Rave, a remote system featuring advanced capabilities in both electronic data capture and clinical data management. Implemented via the Clinic Trials Management Systems (CTMS) project in 2010, the Medidata Rave system is compliant with 21 CFR (Code of Federal Regulations) Part 11 FDA (Food and Drug Administration) requirements. Edit checks, electronic queries, and audit trails are built into the system to ensure accurate and complete data collection and security.

Data storage and management at the Mayo Clinic is under the direction of Dr. Bailey, with the assistance of a Master’s-level statistician. Data from Medidata Rave will be stored as SAS datasets (in Linux) with accessibility restricted to appropriate study members from the Division of Biomedical Statistics and Informatics in the Department of Health Sciences Research at the Mayo Clinic.

Cognitive data storage and management will occur under the direction of Dr. Gleason, with the assistance of a Master’s-level statistician. Coded data will be stored in an R dataset with restricted access. As noted, the code will not be stored on the UW server, and will not be accessible to University of Wisconsin personnel.

## Quality Assurance

In addition to the quality assurance measures implemented at the data entry point, cognitive and mood data will be audited for outliers and inaccuracies through standard data cleaning procedures.[84]

Each MRI and PET/CT acquired on a study subject scan is rated on image quality using a standardized grading form with electronic data entry into a database at the Mayo Clinic.

Quality control measures in the key imaging portions of the study, including MRI acquisition, post-processing of structural MRI images, and PET/CT acquisition and post-processing of PET/CT images are detailed in [Appendix B](#_Appendix_B:_Brain).

## MRI Image Analysis

**Pre-processing to correct specific artifacts:** Several common forms of image imperfection can degrade the quality of the MR data we will collect. These are intensity in-homogeneity due to B1 receiver non-uniformity; drifts or discontinuities in gradient calibration over time; and gradient non-linearity. Image pre-processing operations designed to correct these effects are applied to each set of images prior to image processing.

**MRI analysis of regional cortical structure:** 3D Magnetization Prepared Rapid Acquisition Gradient-Echo (MPRAGE) developed for the multi-center ADNI study by the Mayo MRI Core team,[85] will be used to provide optimal tissue contrast for brain morphology and is harmonized across Siemens, GE and Phillips scanners. We will investigate the AD-signature region of interest (ROI) that we previously identified in clinically diagnosed and autopsy-confirmed AD patients and tested for diagnostic reliability and accuracy.[86] We will investigate both hippocampus and dorsolateral prefrontal cortex volumes because preservation of these structures have been observed after estadiol treatment in ovariectomized animal models.[87] Furthermore, the **preliminary data in Figure 6** suggest that dorsolateral prefrontal cortex volume is preserved in the tE2 group compared to placebo seven years after starting mHT in the KEEPS MRI sub-sample. We will use both publically available FreeSurfer 5.3[88, 89] and an in-house atlas-based ROIs for the analysis of regional volumes adjusted for total intracranial volume (TIV). In addition, we will conduct a non-hypothesis-based voxel-based morphometry (VBM) analysis using Statistical Parametric Mapping 12 (SPM12),[90] to explore regional differences between the mHT and placebo groups in the entire brain using an unbiased approach.

**Segmentation and Quantification of White Matter Hyperintensities (WMH) and identification of infarcts:** Fluid attenuated inversion recovery (FLAIR) with TR/TE/TI = 6000/390/2100 ms with a 800 ms long turbo spin echo readout train, 750 Hz/pixel bandwidth with 3 mm interleaved images of the whole head will be used. WMH volumes are measured using a semi-automated segmentation algorithm as previously described.[91] Briefly, FLAIR and MPRAGE images are co-registered, and the MPRAGE segmentation on SPM12 is used to create a WM mask to reduce false positives on the WMH segmentation from FLAIR. WMH is segmented using an automated slice-based seed initialization and region growing method. The segmented WMH voxels are multiplied with the WM mask and WMH masks are inspected and edited by a trained analyst (blinded to the treatment status) in order to exclude artifacts from the WMH volume. Infarcts will be identified by the same analyst and confirmed by Dr. Kantarci. The hyperintensities on FLAIR images associated with infarcts are marked and are re-classified as hyperintensities associated with cortical or subcortical infarcts. Hyperintensity associated with infarcts are not included in the WMH volume of individual subjects due to pathophysiologic differences between the two lesions.[92] We do not expect a significant number of infarcts in this cohort.

**Diffusion Tensor Imaging (DTI):** 2D single-shot gradient echo sequence with TR/TE = 6600/86 ms, a 128 x 128 base matrix for 240 x 256 mm FOV, 60 contiguous 2 mm slices yielding 2.2 x 2.2 mm in-plane resolution is used for DTI. Diffusion-weighting gradients will be applied along 48 directions with b=1000 m^2^/s and 6 non–diffusion T2 volumes (b = 0 m^2^/s). This sequence was developed for the multi-center LEFFTDS and ARTFL studies by the Mayo Clinic team led by Dr. Kantarci and is harmonized across scanners in participating sites. We recently tested and validated a method to process DTI scans and analyze fractional anisotropy (FA) maps and demonstrated that improved DTI registration outperforms Tract-Based Spatial Statistics.[93] We will conduct non-hypothesis-based voxel-based analysis of FA to explore the WM diffusivity differences between each of the mHT and placebo groups in the entire white matter as previously described.[94]

**Resting state (Rs)-fMRI:** A T2*-weighted gradient echo–echo planar sequence with TR/TE = 2000/27 ms, flip angle 80°; FOV = 230 x 230 mm; matrix size: 92 x 92; 3 mm slices with 2.5 x 2.5 mm in-plane resolution will be used. Subjects are instructed to remain awake with their eyes closed. This sequence was developed for the multi-center LEFFTDS and ARTFL studies by the Mayo Clinic team led by Dr. Kantarci and is harmonized across scanners. The images will be realigned correcting for head motion, and unwarped correcting for susceptibility-by-movement interactions, slice-time corrected, co-registered to T1-weighted image, normalized and smoothed using SPM12. After processing, we will examine connectivity within the default mode network using a seed-based approach, by using the BOLD time series for specific regions as a covariate of interest for whole brain regression analyses.[95, 96] Each of the mHT group maps will be compared to placebo groups with SPM12.

## PET/CT Image Analysis

For all image acquisitions, attenuation correction will use either CT or PET transmission data, and reconstruction will use site-specific algorithms and the PET imaging. After anonymization, data will be electronically transferred to the Mayo Clinic secure servers. Quality control procedures will include checks to assure that the protocol has been followed, checking for full brain coverage, and motion assessment across temporal frames. The sequence of temporal frames are co-registered to the first frame of each scan, and both a dynamic image set, as well as a single averaged-frame image set are produced. Quantitative analysis is performed using the fully automated image processing pipeline, previously described in detail.[97] Briefly, a cortical global Aβ PET standardized uptake value ratio (SUVR) is obtained by combining the prefrontal, orbitofrontal, parietal, temporal, anterior cingulate, and posterior cingulate/precuneus ROI values normalized by the cerebellar ROI of an in-house atlas.

## Training

Trained, certified technicians, who perform the tests also on a clinical basis, will be administering neurocognitive tests, brain MRI, and brain PET/CT.

## Quality Control Committee

Quality control for each test is maintained by the investigative team overseeing that test. Data quality is reviewed by Mayo Clinic and the Principal Investigators for each project.

## Metrics

See [Section 10.3](#_Quality_Assurance) above.

## Protocol Deviations

Any protocol deviations will be documented in Medidata Rave and reviewed by the KEEPS Continuation Executive Committee quarterly throughout the duration of the study.

## Monitoring

Protocol compliance, consent forms, case report forms, timely entry of data, and quality of data will be monitored quarterly by Kent Bailey, Ph.D., assisted by study coordinators and master’s statistician.

# Participant Rights and Confidentiality

## Institutional Review Board (IRB) Review

This protocol, informed consent document, patient contact materials, and any subsequent modifications will be reviewed and approved by the Mayo Clinic IRB responsible for oversight of the study.

## Informed Consent Forms

Informed consent will be obtained in person (between study staff and study candidate) and a signed consent form is required for participation. All participants in KEEPS were English speaking and literate. The consent form describes the purpose of the study, study procedures, and the risks and benefits of participation. The consent form also describes that participation in the study is voluntary and that participants can withdraw at any time. Participants will receive a copy of their consent form for their personal records.

## Participant Confidentiality

Data generated by the research study will be kept strictly confidential. Databases and study documents with identifiers will be kept on a secure Mayo network drive accessible only to a subset of the study team. HIPPA requirements will be followed. Subjects will be assigned code numbers. Study identification numbers will be retained from the KEEPS assignment so as to be able to link all longitudinal data from each participant. These identification numbers are not linked to medical records. All data will be reported in aggregate. Subjects will be told prior to this study that their participation is voluntary. Although we do not anticipate that the subjects will experience discomfort in participating, they can choose not to answer any questions that make them uncomfortable. Information will not be released without written permission of the participant.

## Study Discontinuation

The study may be discontinued at any time by the IRB, the NIA, the OHRP, the FDA, or other government agencies as part of their duties to ensure that research participants are protected.

# Committees

The Co-PIs, Dr. Kejal Kantarci and Dr. Carey Gleason, will be responsible for planning, reviewing, coordinating, and directing the project. They will direct and oversee all scientific management, administrative, and financial aspects of the program. They will make changes to the program as deemed necessary in consultation with the Executive Committee that consists of site-PIs and meets once a month over tele-conferences.

## Day-to-Day Management at Mayo Clinic

Ekta Kappor, M.D., who is a women’s health specialist is responsible for recruitment, screening, consent and enrollment of study participants, coordination and completion of clinical research testing. Julie Fields, Ph.D., L.P. is responsible for management and oversight of neurocognitive testing. Kent Bailey, Ph.D. is responsible for database management and statistical analyses.

Site-PIs are responsible for the day-to-day administration of the protocol at their sites, including data collection, and transfer.

## External Advisory Committee

An External Advisory Committee will review productivity, allocation of funds, and other issues that may arise in relationship to progress. This committee may recommend changes needed for the research direction/emphasis. Members of this committee were selected specifically for their leadership activities, for their clinical and research activities related to the overarching theme of the KEEPS Continuation. Members of this committee include:

S. Mitchell Harman, M.D. (chair-PI of the original KEEPS);

Susan Resnick, Ph.D.;

Eric Reiman, M.D.;

Clifford Jack, M.D.;

Pauline Maki, Ph.D.;

Nannette Santoro, M.D.; and

Frederick Naftolin, M.D.

The advisory committee will meet over teleconferences and an in-person meeting with key personnel

# Publication of Research Findings

Publication of the results of this trial will be governed by the policies and procedures developed by the Executive Committee. All publications will be made public through NIHMS.

# Supplements/Appendices

[Appendix A](#_Appendix_A:_Laboratory): Laboratory Tests

[Appendix B](#_Appendix_B:_Brain): Brain MRI and PET/CT Acquisition and Post-Processing

[Appendix C](#_Appendix_C:_Pregnancy): Pregnancy Questionnaire

# References

1. Seshadri S, Wolf PA, Beiser A, Au R, McNulty K, White R, et al. Lifetime risk of dementia and Alzheimer's disease - The impact of mortality on risk estimates in the Framingham Study. Neurology. 1997;49(6):1498-504.

2. Sperling RA, Rentz DM, Johnson KA, Karlawish J, Donohue M, Salmon DP, et al. The A4 study: stopping AD before symptoms begin? Science translational medicine. 2014;6(228):228fs13.

3. Sperling RA, Jack CR, Jr., Aisen PS. Testing the right target and right drug at the right stage. Science translational medicine. 2011;3(111):111cm33.

4. Manson JE. Overview of Menopause. Menopause practice: A clinician’s guide. 4th Edition ed. Mayfield Heights, OH: North American Menopause Society; 2010.

5. Harman SM, Brinton EA, Cedars M, Lobo R, Manson JE, Merriam GR, et al. KEEPS: The Kronos Early Estrogen Prevention Study. Climacteric. 2005;8(1):3-12.

6. Shumaker SA, Reboussin BA, Espeland MA, Rapp SR, McBee WL, Dailey M, et al. The Women's Health Initiative Memory Study (WHIMS): a trial of the effect of estrogen therapy in preventing and slowing the progression of dementia. Control Clin Trials. 1998;19(6):604-21.

7. Espeland MA, Shumaker SA, Leng I, Manson JE, Brown CM, LeBlanc ES, et al. Long-term effects on cognitive function of postmenopausal hormone therapy prescribed to women aged 50 to 55 years. JAMA Intern Med. 2013;173(15):1429-36.

8. LeBlanc ES, Janowsky J, Chan BK, Nelson HD. Hormone replacement therapy and cognition: systematic review and meta-analysis. Jama. 2001;285(11):1489-99.

9. Sherwin BB. Estrogen and memory in women: how can we reconcile the findings? Horm Behav. 2005;47(3):371-5.

10. Waring SC, Rocca WA, Petersen RC, O'Brien PC, Tangalos EG, Kokmen E. Postmenopausal estrogen replacement therapy and risk of AD: a population-based study. Neurology. 1999;52(5):965-70.

11. Whitmer RA, Quesenberry CP, Zhou J, Yaffe K. Timing of hormone therapy and dementia: the critical window theory revisited. Ann Neurol. 2011;69(1):163-9.

12. Yaffe K, Sawaya G, Lieberburg I, Grady D. Estrogen therapy in postmenopausal women: Effects on cognitive function and dementia. Jama. 1998;279:688-95.

13. Zandi PP, Carlson MC, Plassman BL, Welsh-Bohmer KA, Mayer LS, Steffens DC, et al. Hormone replacement therapy and incidence of Alzheimer disease in older women: the Cache County Study. Jama. 2002;288(17):2123-9.

14. Rocca WA, Grossardt BR, Shuster LT. Oophorectomy, estrogen, and dementia: a 2014 update. Molecular and cellular endocrinology. 2014;389(1-2):7-12.

15. Rossouw JE, Anderson GL, Prentice RL, LaCroix AZ, Kooperberg C, Stefanick ML, et al. Risks and benefits of estrogen plus progestin in healthy postmenopausal women: principal results From the Women's Health Initiative randomized controlled trial. Jama. 2002;288(3):321-33.

16. Rapp SR, Espeland MA, Shumaker SA, Henderson VW, Brunner RL, Manson JE, et al. Effect of estrogen plus progestin on global cognitive function in postmenopausal women: the Women's Health Initiative Memory Study: a randomized controlled trial. Jama. 2003;289(20):2663-72.

17. Shumaker SA, Legault C, Kuller L, Rapp SR, Thal L, Lane DS, et al. Conjugated equine estrogens and incidence of probable dementia and mild cognitive impairment in postmenopausal women: Women's Health Initiative Memory Study. 2004;291(24):2947-58.

18. Resnick SM, Henderson VW. Hormone therapy and risk of Alzheimer disease: a critical time. Jama. 2002;288(17):2170-2.

19. Jack CR, Jr., Knopman DS, Jagust WJ, Petersen RC, Weiner MW, Aisen PS, et al. Tracking pathophysiological processes in Alzheimer's disease: an updated hypothetical model of dynamic biomarkers. Lancet Neurol. 2013;12(2):207-16.

20. Reiman EM, Langbaum JB, Fleisher AS, Caselli RJ, Chen K, Ayutyanont N, et al. Alzheimer's Prevention Initiative: a plan to accelerate the evaluation of presymptomatic treatments. J Alzheimers Dis. 2011;26 Suppl 3:321-9.

21. Mikkola TS, Savolainen-Peltonen H, Tuomikoski P, Hoti F, Vattulainen P, Gissler M, et al. Lower Death Risk for Vascular Dementia than for Alzheimer's Disease with Postmenopausal Hormone Therapy Users. J Clin Endocrinol Metab. 2016:jc20163590.

22. McEwen B. Estrogen actions throughout the brain. Recent Prog Horm Res. 2002;57:357-84.

23. McAsey ME, Cady C, Jackson LM, Li M, Randall S, Nathan BP, et al. Time course of response to estradiol replacement in ovariectomized mice: brain apolipoprotein E and synaptophysin transiently increase and glial fibrillary acidic protein is suppressed. Exp Neurol. 2006;197(1):197-205.

24. Thomas T, Bryant M, Clark L, Garces A, Rhodin J. Estrogen and raloxifene activities on amyloid-beta-induced inflammatory reaction. Microvasc Res. 2001;61(1):28-39.

25. Li R, Shen Y, Yang LB, Lue LF, Finch C, Rogers J. Estrogen enhances uptake of amyloid beta-protein by microglia derived from the human cortex. J Neurochem. 2000;75(4):1447-54.

26. Greenfield JP, Leung LW, Cai D, Kaasik K, Gross RS, Rodriguez-Boulan E, et al. Estrogen lowers Alzheimer beta-amyloid generation by stimulating trans-Golgi network vesicle biogenesis. J Biol Chem. 2002;277(14):12128-36.

27. Hao J, Janssen WG, Tang Y, Roberts JA, McKay H, Lasley B, et al. Estrogen increases the number of spinophilin-immunoreactive spines in the hippocampus of young and aged female rhesus monkeys. J Comp Neurol. 2003;465(4):540-50.

28. Sakamoto H, Mezaki Y, Shikimi H, Ukena K, Tsutsui K. Dendritic growth and spine formation in response to estrogen in the developing Purkinje cell. Endocrinology. 2003;144(10):4466-77.

29. Tang Y, Janssen WG, Hao J, Roberts JA, McKay H, Lasley B, et al. Estrogen replacement increases spinophilin-immunoreactive spine number in the prefrontal cortex of female rhesus monkeys. Cereb Cortex. 2004;14(2):215-23.

30. Maki PM. Estrogen effects on the hippocampus and frontal lobes. Int J Fertil Womens Med. 2005;50(2):67-71.

31. Prokai L, Prokai-Tatrai K, Perjesi P, Zharikova AD, Perez EJ, Liu R, et al. Quinol-based cyclic antioxidant mechanism in estrogen neuroprotection. Proc Natl Acad Sci U S A. 2003;100(20):11741-6.

32. Hodis HN, Mack WJ, Shoupe D, Azen SP, Stanczyk FZ, Hwang-Levine J, et al. Methods and baseline cardiovascular data from the Early versus Late Intervention Trial with Estradiol testing the menopausal hormone timing hypothesis. Menopause. 2015;22(4):391-401.

33. Hodis HN, Mack WJ, Henderson VW, Shoupe D, Budoff MJ, Hwang-Levine J, et al. Vascular Effects of Early versus Late Postmenopausal Treatment with Estradiol. N Engl J Med. 2016;374(13):1221-31.

34. Brinton RD. Investigative models for determining hormone therapy-induced outcomes in brain: evidence in support of a healthy cell bias of estrogen action. Ann N Y Acad Sci. 2005;1052:57-74.

35. Chen S, Nilsen J, Brinton RD. Dose and temporal pattern of estrogen exposure determines neuroprotective outcome in hippocampal neurons: therapeutic implications. Endocrinology. 2006;147(11):5303-13.

36. Nilsen J, Brinton RD. Mitochondria as therapeutic targets of estrogen action in the central nervous system. Curr Drug Targets CNS Neurol Disord. 2004;3(4):297-313.

37. Nilsen J, Chen S, Irwin RW, Iwamoto S, Brinton RD. Estrogen protects neuronal cells from amyloid beta-induced apoptosis via regulation of mitochondrial proteins and function. BMC Neurosci. 2006;7:74.

38. Simpkins JW, Wang J, Wang X, Perez E, Prokai L, Dykens JA. Mitochondria play a central role in estrogen-induced neuroprotection. Curr Drug Targets CNS Neurol Disord. 2005;4(1):69-83.

39. Asthana S, Brinton RD, Henderson VW, McEwen BS, Morrison JH, Schmidt PJ. Frontiers proposal. National Institute on Aging "bench to bedside: estrogen as a case study". Age (Dordr). 2009;31(3):199-210.

40. Espeland MA, Brinton RD, Manson JE, Yaffe K, Hugenschmidt C, Vaughan L, et al. Postmenopausal hormone therapy, type 2 diabetes mellitus, and brain volumes. Neurology. 2015;85(13):1131-8.

41. Maki PM. A systematic review of clinical trials of hormone therapy on cognitive function: effects of age at initiation and progestin use. Ann N Y Acad Sci. 2005;1052:182-97.

42. Shumaker SA, Legault C, Rapp SR, Thal L, Wallace RB, Ockene JK, et al. Estrogen plus progestin and the incidence of dementia and mild cognitive impairment in postmenopausal women: the Women's Health Initiative Memory Study: a randomized controlled trial. Jama. 2003;289(20):2651-62.

43. Espeland MA, Rapp SR, Shumaker SA, Brunner R, Manson JE, Sherwin BB, et al. Conjugated equine estrogens and global cognitive function in postmenopausal women: Women's Health Initiative Memory Study. Jama. 2004;291(24):2959-68.

44. Resnick SM, Espeland MA, An Y, Maki PM, Coker LH, Jackson R, et al. Effects of conjugated equine estrogens on cognition and affect in postmenopausal women with prior hysterectomy. J Clin Endocrinol Metab. 2009;94(11):4152-61.

45. Resnick SM, Maki PM, Rapp SR, Espeland MA, Brunner R, Coker LH, et al. Effects of combination estrogen plus progestin hormone treatment on cognition and affect. J Clin Endocrinol Metab. 2006;91(5):1802-10.

46. Resnick SM, Espeland MA, Jaramillo SA, Hirsch C, Stefanick ML, Murray AM, et al. Postmenopausal hormone therapy and regional brain volumes: the WHIMS-MRI Study. Neurology. 2009;72(2):135-42.

47. Espeland MA, Pettinger M, Falkner KL, Shumaker SA, Limacher M, Thomas F, et al. Demographic and health factors associated with enrollment in posttrial studies: The women's health initiative hormone therapy trials. Clin Trials. 2013;10(3):463-72.

48. Henderson VW, St John JA, Hodis HN, McCleary CA, Stanczyk FZ, Shoupe D, et al. Cognitive effects of estradiol after menopause: A randomized trial of the timing hypothesis. Neurology. 2016;87(7):699-708.

49. Bagger YZ, Tanko LB, Alexandersen P, Qin G, Christiansen C. Early postmenopausal hormone therapy may prevent cognitive impairment later in life. Menopause. 2005;12(1):12-7.

50. Design of the Women's Health Initiative clinical trial and observational study. The Women's Health Initiative Study Group. Control Clin Trials. 1998;19(1):61-109.

51. Clark CM, Schneider JA, Bedell BJ, Beach TG, Bilker WB, Mintun MA, et al. Use of florbetapir-PET for imaging beta-amyloid pathology. Jama. 2011;305(3):275-83.

52. Sperling RA, Aisen PS, Beckett LA, Bennett DA, Craft S, Fagan AM, et al. Toward defining the preclinical stages of Alzheimer's disease: recommendations from the National Institute on Aging-Alzheimer's Association workgroups on diagnostic guidelines for Alzheimer's disease. Alzheimers Dement. 2011;7(3):280-92.

53. Dubois B, Hampel H, Feldman HH, Scheltens P, Aisen P, Andrieu S, et al. Preclinical Alzheimer's disease: Definition, natural history, and diagnostic criteria. Alzheimers Dement. 2016;12(3):292-323.

54. Morris JC, Roe CM, Xiong C, Fagan AM, Goate AM, Holtzman DM, et al. APOE predicts amyloid-beta but not tau Alzheimer pathology in cognitively normal aging. Ann Neurol. 2010;67(1):122-31.

55. Payami H, Zareparsi S, Montee KR, Sexton GJ, Kaye JA, Bird TD, et al. Gender difference in apolipoprotein E-associated risk for familial Alzheimer disease: a possible clue to the higher incidence of Alzheimer disease in women. American journal of human genetics. 1996;58(4):803-11.

56. Rocca WA, Mielke MM, Vemuri P, Miller VM. Sex and gender differences in the causes of dementia: a narrative review. Maturitas. 2014;79(2):196-201.

57. Altmann A, Tian L, Henderson VW, Greicius MD. Sex modifies the APOE-related risk of developing Alzheimer disease. Ann Neurol. 2014;75(4):563-73.

58. Corder EH, Ghebremedhin E, Taylor MG, Thal DR, Ohm TG, Braak H. The biphasic relationship between regional brain senile plaque and neurofibrillary tangle distributions: modification by age, sex, and APOE polymorphism. Annals of the New York Academy of Sciences. 2004;1019:24-8.

59. Resnick SM, Bilgel M, Moghekar A, An Y, Cai Q, Wang MC, et al. Changes in Abeta biomarkers and associations with APOE genotype in 2 longitudinal cohorts. Neurobiol Aging. 2015;36(8):2333-9.

60. Kantarci K, Lowe VJ, Lesnick TG, Tosakulwong N, Bailey KR, Fields JA, et al. Early Postmenopausal Transdermal 17beta-Estradiol Therapy and Amyloid-beta Deposition. J Alzheimers Dis. 2016;53(2):547-56.

61. Greenberg DL, Payne ME, MacFall JR, Provenzale JM, Steffens DC, Krishnan RR. Differences in brain volumes among males and female hormone-therapy users and nonusers. Psychiatry Res. 2006;147(2-3):127-34.

62. Eberling JL, Wu C, Haan MN, Mungas D, Buonocore M, Jagust WJ. Preliminary evidence that estrogen protects against age-related hippocampal atrophy. Neurobiol Aging. 2003;24(5):725-32.

63. Boccardi M, Ghidoni R, Govoni S, Testa C, Benussi L, Bonetti M, et al. Effects of hormone therapy on brain morphology of healthy postmenopausal women: a Voxel-based morphometry study. Menopause. 2006;13(4):584-91.

64. Lord C, Buss C, Lupien SJ, Pruessner JC. Hippocampal volumes are larger in postmenopausal women using estrogen therapy compared to past users, never users and men: a possible window of opportunity effect. Neurobiol Aging. 2008;29(1):95-101.

65. Erickson KI, Colcombe SJ, Raz N, Korol DL, Scalf P, Webb A, et al. Selective sparing of brain tissue in postmenopausal women receiving hormone replacement therapy. Neurobiol Aging. 2005;26(8):1205-13.

66. Ha DM, Xu J, Janowsky JS. Preliminary evidence that long-term estrogen use reduces white matter loss in aging. Neurobiol Aging. 2007;28(12):1936-40.

67. Coker LH, Hogan PE, Bryan NR, Kuller LH, Margolis KL, Bettermann K, et al. Postmenopausal hormone therapy and subclinical cerebrovascular disease: the WHIMS-MRI Study. Neurology. 2009;72(2):125-34.

68. Espeland MA, Tindle HA, Bushnell CA, Jaramillo SA, Kuller LH, Margolis KL, et al. Brain volumes, cognitive impairment, and conjugated equine estrogens. J Gerontol A Biol Sci Med Sci. 2009;64(12):1243-50.

69. Kuller LH, Margolis KL, Gaussoin SA, Bryan NR, Kerwin D, Limacher M, et al. Relationship of hypertension, blood pressure, and blood pressure control with white matter abnormalities in the Women's Health Initiative Memory Study (WHIMS)-MRI trial. J Clin Hypertens (Greenwich). 2010;12(3):203-12.

70. Jagust WJ, Zheng L, Harvey DJ, Mack WJ, Vinters HV, Weiner MW, et al. Neuropathological basis of magnetic resonance images in aging and dementia. Ann Neurol. 2008;63(1):72-80.

71. Seliger SL, Longstreth WT, Jr., Katz R, Manolio T, Fried LF, Shlipak M, et al. Cystatin C and subclinical brain infarction. J Am Soc Nephrol. 2005;16(12):3721-7.

72. Schmidt R, Ropele S, Enzinger C, Petrovic K, Smith S, Schmidt H, et al. White matter lesion progression, brain atrophy, and cognitive decline: the Austrian stroke prevention study. Ann Neurol. 2005;58(4):610-6.

73. Vermeer SE, Prins ND, den Heijer T, Hofman A, Koudstaal PJ, Breteler MM. Silent brain infarcts and the risk of dementia and cognitive decline. N Engl J Med. 2003;348(13):1215-22.

74. DeCarli C, Miller BL, Swan GE, Reed T, Wolf PA, Carmelli D. Cerebrovascular and brain morphologic correlates of mild cognitive impairment in the National Heart, Lung, and Blood Institute Twin Study. Arch Neurol. 2001;58(4):643-7.

75. Luchsinger J, Brickman A, Reitz C, Schupf N, Manly J, Tang M, et al. Cerebrovascular disease in mild cognitive impairment. Alzheimers Dement (Amst). 2008;4(4(2)):T131.

76. Kantarci K, Petersen RC, Przybelski SA, Weigand SD, Shiung MM, Whitwell JL, et al. Hippocampal volumes, proton magnetic resonance spectroscopy metabolites, and cerebrovascular disease in mild cognitive impairment subtypes. Arch Neurol. 2008;65(12):1621-8.

77. Harman SM, Black DM, Naftolin F, Brinton EA, Budoff MJ, Cedars MI, et al. Arterial imaging outcomes and cardiovascular risk factors in recently menopausal women: a randomized trial. Ann Intern Med. 2014;161(4):249-60.

78. Manson JE, Allison MA, Rossouw JE, Carr JJ, Langer RD, Hsia J, et al. Estrogen therapy and coronary-artery calcification. N Engl J Med. 2007;356(25):2591-602.

79. Hodis HN, Mack WJ, Lobo RA, Shoupe D, Sevanian A, Mahrer PR, et al. Estrogen in the prevention of atherosclerosis. A randomized, double-blind, placebo-controlled trial. Ann Intern Med. 2001;135(11):939-53.

80. Albert MS, DeKosky ST, Dickson D, Dubois B, Feldman HH, Fox NC, et al. The diagnosis of mild cognitive impairment due to Alzheimer's disease: recommendations from the National Institute on Aging-Alzheimer's Association workgroups on diagnostic guidelines for Alzheimer's disease. Alzheimers Dement. 2011;7(3):270-9.

81. Gleason CE, Dowling NM, Wharton W, Manson JE, Miller VM, Atwood CS, et al. Effects of Hormone Therapy on Cognition and Mood in Recently Postmenopausal Women: Findings from the Randomized, Controlled KEEPS-Cognitive and Affective Study. PLOS Medicine. 2015;12(6):e1001833; discussion e.

82. Dowling NM, Gleason CE, Manson JE, Hodis HN, Miller VM, Brinton EA, et al. Characterization of vascular disease risk in postmenopausal women and its association with cognitive performance. PLoS One. 2013;8(7):e68741.

83. Roberts RO, Geda YE, Knopman DS, Cha RH, Pankratz VS, Boeve BF, et al. The Mayo Clinic Study of Aging: design and sampling, participation, baseline measures and sample characteristics. Neuroepidemiology. 2008;30(1):58-69.

84. Ganti V, Das Sarma A. Data cleaning : a practical perspective. San Rafael, California: Morgan & Claypool,; 2013. Available from: <http://www.morganclaypool.com/doi/abs/10.2200/S00523ED1V01Y201307DTM036> Available through Synthesis Digital Library of Engineering and Computer Science.

85. Jack CR, Jr., Barnes J, Bernstein MA, Borowski BJ, Brewer J, Clegg S, et al. Magnetic resonance imaging in Alzheimer's Disease Neuroimaging Initiative 2. Alzheimers Dement. 2015;11(7):740-56.

86. Schwarz CG, Gunter JL, Wiste HJ, Przybelski SA, Weigand SD, Ward CP, et al. A large-scale comparison of cortical thickness and volume methods for measuring Alzheimer’s Disease Severity. Neuroimage Clinical in press. 2016.

87. Hara Y, Waters EM, McEwen BS, Morrison JH. Estrogen Effects on Cognitive and Synaptic Health Over the Lifecourse. Physiol Rev. 2015;95(3):785-807.

88. Fischl B, Sereno MI, Dale AM. Cortical surface-based analysis. II: Inflation, flattening, and a surface-based coordinate system. Neuroimage. 1999;9(2):195-207.

89. Dale AM, Fischl B, Sereno MI. Cortical surface-based analysis. I. Segmentation and surface reconstruction. Neuroimage. 1999;9(2):179-94.

90. Ashburner J, Friston KJ. Unified segmentation. Neuroimage. 2005;26(3):839-51.

91. Raz L, Jayachandran M, Tosakulwong N, Lesnick TG, Wille SM, Murphy MC, et al. Thrombogenic microvesicles and white matter hyperintensities in postmenopausal women. Neurology. 2013;80(10):911-8.

92. Kantarci K, Weigand SD, Przybelski SA, Shiung MM, Whitwell JL, Negash S, et al. Risk of dementia in MCI: combined effect of cerebrovascular disease, volumetric MRI, and 1H MRS. Neurology. 2009;72(17):1519-25.

93. Schwarz CG, Reid RI, Gunter JL, Senjem ML, Przybelski SA, Zuk SM, et al. Improved DTI registration allows voxel-based analysis that outperforms Tract-Based Spatial Statistics. NeuroImage. 2014.

94. Nedelska Z, Schwarz CG, Boeve BF, Lowe VJ, Reid RI, Przybelski SA, et al. White matter integrity in dementia with Lewy bodies: a voxel-based analysis of diffusion tensor imaging. Neurobiol Aging. 2015;36(6):2010-7.

95. Jones D, Graff-Radford J, Przybelski S, Knopman DS, Petersen RC, Jack CR, Jr., et al., editors. The pattern of posterior default mode network failure is unique for dementia with Lewy bodies and Alzheimer’s disease dementia. International Dementia with Lewy Bodies Conference; 2015; Fort Lauderdale, FL: American journal of Neurodegenerative Diseases

96. Kantarci K, Jones DT, Boeve BF, Vemuri P, Machulda M, Gunter J, et al., editors. Effects of Beta-Amyloid Load on Task-free fMRI Abnormalities in Dementia with Lewy Bodies. Alzheimer's Association International Conference; 2012; Vancouver, BC.

97. Kantarci K, Gunter JL, Tosakulwong N, Weigand SD, Senjem MS, Petersen RC, et al. Focal hemosiderin deposits and beta-amyloid load in the ADNI cohort. Alzheimers Dement. 2013;9(5 Suppl):S116-23.

# Appendix A: Laboratory Tests

| **Variables** | **Label** |
| --- | --- |
| T_Chol | Total cholesterol (mg/dL) |
| LDL | LDL (low density lipoprotein) cholesterol (mg/dL) |
| HDL | HDL (high density lipoprotein) cholesterol (mg/dL) |
| TG | Triglycerides (mg/dL) |
| FBG | Fasting glucose |
| Insulin | Insulin |
| A1C | Hemoglobin A1C |
| TSH | Thyroid stimulating hormone |
| VitB1 | Vitamin B12 |

# Appendix B: Brain MRI and PET/CT Acquisition and Post-Processing

**PET Acquisition**

Analysis will be performed with SPM12 and PMOD (version 2.75; PMOD Technologies, Zurich, CH). The individual frames of the F-18 Florbetapir dynamic series will be realigned if motion is detected and then a mean image created. PET quantitative image analysis is performed using the fully automated image processing pipeline which has previously been described in detail.(87) Briefly, the method includes registering PET images to 3D-MPRAGE for gray matter (GM) sharpening on SPM5. F-18 Florbetapir PET cortical ratio images are calculated by dividing each PET GM voxel value by the median value in the cerebellar GM region in the subject’s MRI space. The global cortical F-18 Florbetapir retention is calculated by the median value of the F-18 Florbetapir PET GM ratio from among all voxels in the bilateral parietal, posterior cingulate, precuneus, temporal, prefrontal, orbitofrontal, and anterior cingulate GM regions as defined in the in-house modified anatomical labeling atlas where the average is weighted by ROI size.(88)

**Longitudinal voxel-based morphometry**

4D voxel-wise maps of gray matter change over time

Voxel-based morphometry (VBM), within the statistical parametric mapping (SPM12) suite (89), will be used to evaluate brain morphometry on a voxel-wise basis.

First, a custom template and tissue probability maps (TPMs) are created in SPM12 using the MPRAGE MRI scans from all subjects in the analysis. The custom template and TPMs are created by first normalizing and segmenting all MPRAGE scans using the unified segmentation model in SPM12 with the standard Montreal Neurological Institute (MNI) template and TPMs, followed by a **clean-up step which uses a hidden Markov random field (HMRF)** model to increase the accuracy of the individual subject TPMs, and finally averaging the normalized subject TPMs.(90)

We have implemented tensor based morphometry (TBM) in SPM12, which measures voxel-wise rates of decline of gray matter (GM) density captured by serial MRI studies.(91) The basic steps of TBM are:

- 1. Computing deformation from late to early images
  2. Creating soft-mean of early and deformed-late images
  3. Creating image of the deformation’s Jacobian determinants
  4. Normalizing and segmenting the soft-mean image, applying the normalization to the Jacobian image
  5. Multiplying normalized Jacobian with segmented GM
  6. Modulating the normalized early and late GM images
  7. Smoothing the early and late GM images with an 8 mm kernel
  8. Performing statistical comparisons between groups of smoothed early and late GM images, to generate maps showing differences in GM density decline over time between groups within the general linear model framework of SPM (p<0.05) corrected for multiple comparisons using family-wise error correction

# Appendix C: Pregnancy Questionnaire

1. Are you currently taking prescription medication to lower your blood pressure?
2. Yes
3. No
4. Do not know
5. Are you currently taking prescription medication for diabetes?
6. Yes
7. No
8. Do not know
9. Have you had at least one pregnancy that lasted more than 6 months?
10. Yes
11. No
12. Before your first pregnancy, did you have (circle all that apply):
    1. Protein in the urine
    2. Seizures or convulsions
    3. High blood pressure (hypertension)
    4. Diabetes
13. During any of these pregnancies (which lasted more than 6 months), did a physician ever tell you that you had high blood pressure or hypertension?
14. Yes (circle i. or ii., then go to question 6)
15. ln one pregnancy only
16. ln more than one pregnancy
17. No (go to question 7)
18. During any of the pregnancies in which you developed hypertension, did you have:
19. Protein in the urine
20. Seizures or convulsions
21. Preeclampsia, eclampsia, or toxemia of pregnancy
22. During any of your pregnancies, did you have preeclampsia, eclampsia, or toxemia?
23. Yes
24. ln one pregnancy only
25. ln more than one pregnancy
26. No
27. During any of these pregnancies (which lasted more than 6 months), did a physician ever tell you that you had diabetes or gestational diabetes?
28. Yes
29. ln one pregnancy only
30. ln more than one pregnancy
31. No
32. During any of these pregnancies (which lasted more than 6 months), did a physician ever tell you that your delivery was premature (preterm), occurring before 37 weeks of gestation?
    1. Yes
33. ln one pregnancy only
34. ln more than one pregnancy
    1. No
35. During any of these pregnancies (which lasted more than 6 months), did a physician ever tell you that your baby was of low birth weight?
    1. Yes
    2. In one pregnancy only
    3. In more than one pregnancy
    4. No
