# Supplementary material for: Long-term cognitive effects of menopausal hormone therapy: Findings from the KEEPS Continuation Study
Source: PLoS Med. 2024 Nov 21;21(11):e1004435. doi: 10.1371/journal.pmed.1004435 (PMC11581397; doi:10.1371/journal.pmed.1004435)
Supplement: S1 Methods — (DOCX) [file pmed.1004435.s003.docx]

**Supplement**

**S1 Statistical Methods**

*Supplementary Information about Latent Growth Models (LGM) with a Distal Outcome.*

The Latent Growth Model (LGM) with a distal outcome approach was selected over the originally planned linear mixed effect (LME) for several reasons all related to the phenomenon that participants were re-evaluated after 10+ years with no monitoring. Primary among these were, 1) LGMs are better suited for fitting models with a distal outcome [1]; and 2) LGM allows us to observe non-linear changes in cognition, and 3) LGM approach permits us to model change as a random effect [2].

Standard assumptions of the LGM are: (a) the means of all latent variables, error terms, and factors have zero variance; (b) the variances of all latent variables have zero means; (c) the means and variances of latent variables do not covary; and (d) the error variances do not covary with each other or with any variables except the measured variables they directly affect [2].

The LGM model [3, 4] can be expressed as a measurement model (Equation 1)

$Y_{it}=\theta_{0i}+\theta_{1i}\alpha_{t}+\varepsilon_{it}where \varepsilon_{it}\sim N\left( 0,\sigma^{2} \right)$

$Y_{it}$ is the cognitive outcome for person *i* at time *t*, $\theta_{0i}$ represents initial status at time 0 (α = 0), $\theta_{1i}$ represents the curve trajectory or rate of change between data collection time points (Month 0, 18, 36, and 48), and $\varepsilon_{it}$ is a residual term within each time assumed normally and independently distributed with mean 0 and variance $\sigma^{2}$

The structural model (Equation 2) containing the growth parameters representing the individual growth/change scores in Equation 1 can be viewed as outcomes in a between-individual model. These are expressed as:

$$\theta_{oi}=\mu_{0}+\varsigma_{0i} and$$

$\theta_{1i}=\mu_{1}+\varsigma_{1i}$

µ_0_ and µ_1_ represent the population means for initial cognitive status (intercept factor values) and curve trajectory across time (population mean of individual slope factor values), respectively.

$\varsigma_{0i}$ and $\varsigma_{1i}$ represent the deviations of individual *i’s* growth parameters from each of these overall means values. The mean values are referred to as fixed effects and the deviation values are random effects. It is assumed that the deviations are normally distributed;

$\left[ \begin{matrix} \varsigma_{0i} \\ \varsigma_{1i} \end{matrix} \right]\sim N$ $\left( \left[ \begin{matrix} 0 \\ 0 \end{matrix} \right],\left[ \begin{matrix} \sigma_{00}^{2} & \sigma_{01} \\ \sigma_{10} & \sigma_{11}^{2} \end{matrix} \right] \right)$

such that $\sigma_{00}^{2}$ is the variance in initial status as Month 0, $\sigma_{11}^{2}$ is the variance of the rates of change, and $\sigma_{10}$ is the covariance between status in Month 0 and rate of change across time.

The LGM can be extended by including a distal outcome. If we add a distal outcome variable (cognitive status 8-14 years post randomization) the structural model (Equation 2 above) is extended as follows

$$\theta_{Di}=\mu_{D}+\beta_{1}\theta_{0i}+\beta_{2}\theta_{1i}+\varsigma_{Di}$$

where $\theta_{Di}$ is the individual-specific latent factor for the distal outcome, $\mu_{D}$ is the intercept of the distal outcome (the population mean of the individual distal outcome variable values when $\theta_{0i}$and $\theta_{1i}$ are zero. β_1_ and β_2_ are the regression coefficients representing the relations between the LGM and the distal outcome variable, and $\varsigma_{Di}$ represents the individual-specific difference between $\theta_{Di}$and $\mu_{D}$.

All our LGM models controlled for education, age, and APOEe4 carrier status.

The diagrammatic representation of the basic latent growth model with a distal outcome is presented below:


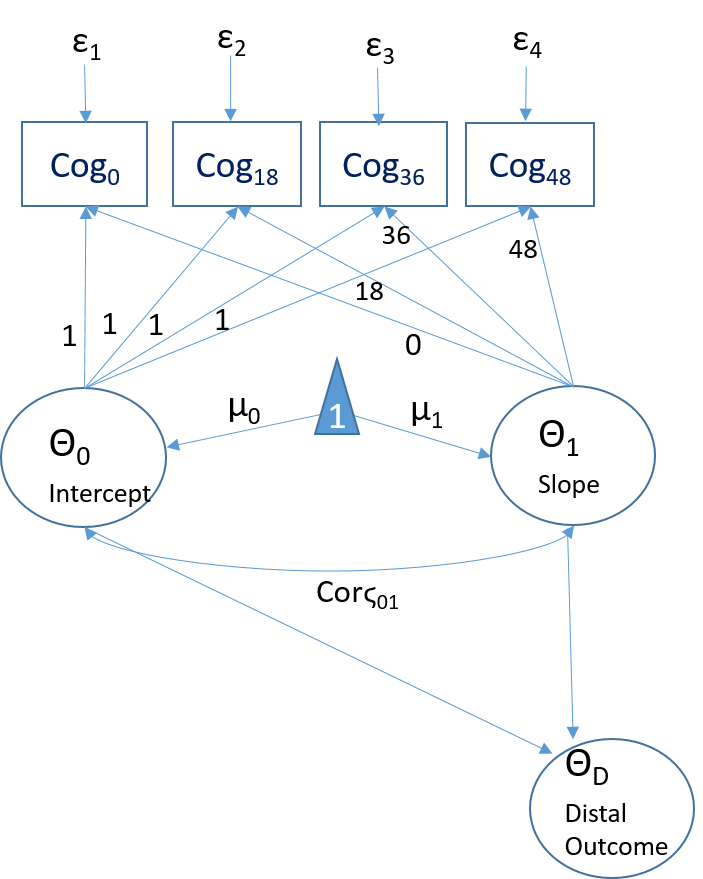


**References**

1. McCormick EM, Curran PJ, Hancock GR. Latent growth factors as predictors of distal outcomes. Psychol Methods. 2024.

2. McNeish D, Matta T. Differentiating between mixed-effects and latent-curve approaches to growth modeling. Behavior Research Methods. 2018;50(4):1398-414.

3. McArdle JJ. Latent curve modeling of longitudinal growth data. Handbook of structural equation modeling. New York, NY, US: The Guilford Press; 2012. p. 547-70.

4. Little TD. Longitudinal structural equation modeling, 2nd ed. New York, NY, US: The Guilford Press; 2024. xxiv, 616-xxiv, p.
