# Supplementary material for: Long-term cognitive effects of menopausal hormone therapy: Findings from the KEEPS Continuation Study
Source: PLoS Med. 2024 Nov 21;21(11):e1004435. doi: 10.1371/journal.pmed.1004435 (PMC11581397; doi:10.1371/journal.pmed.1004435)
Supplement: S1 Table — (DOCX) [file pmed.1004435.s004.docx]

**Supplement**

**S1 Table**: Linear latent growth models for cognitive outcomes showing the association between intercept and slope for cognitive performance during hormone therapy and later cognitive function after excluding n= 40 participants who continue the use of systemic mHT**.**

| Variable | Estimate | S.E. | P-Value | 95% Confidence Intervals |
| --- | --- | --- | --- | --- |
| **Verbal Attention & Executive Function** | | | | |
| Intercept for cognitive performance | 0.379 | 0.059 | **<0.001** | (0.262, 0.495) |
| Slope for cognitive performance | 0.550 | 0.062 | **<0.001** | (0.429, 0.672) |
| *Effect of mHT allocation during KEEPS trial on slope for cognitive performance* |  |  |  |  |
| oCEE | 0.014 | 0.052 | 0.783 | (-0.088, 0.117) |
| tE2 | -0.031 | 0.052 | 0.548 | (-0.132, 0.070) |
| *Effect of mHT allocation during KEEPS trial later cognitive function* |  |  |  |  |
| oCEE | -0.019 | 0.042 | 0.655 | (-0.102, 0.064) |
| tE2 | -0.074 | 0.044 | 0.089 | (-0.160, 0.011) |
| **Fit Indices** | | | | |
| CFI=0.968; SRMR=0.058 | | | | |
| AIC=6319.646; BIC=6435.568 | | | | |
| **Speeded Language & Mental Flexibility** | | | | |
| Intercept for cognitive performance | 0.501 | 0.078 | **<0.001** | (0.349, 0.653) |
| Slope for cognitive Performance | 0.461 | 0.100 | **<0.001** | (0.266, 0.657) |
| *Effect of mHT allocation during KEEPS trial on Slope for Cognitive Performance* |  |  |  |  |
| oCEE | -0.006 | 0.054 | 0.910 | (-0.111, 0.099) |
| tE2 | -0.015 | 0.054 | 0.777 | (-0.120, 0.090) |
| *Effect of mHT allocation during KEEPS trial on later cognitive function* |  |  |  |  |
| oCEE | -0.009 | 0.04 | 0.830 | (-0.088, 0.070) |
| tE2 | -0.048 | 0.042 | 0.259 | (-0.131, 0.035) |
| **Fit Indices** | | | | |
| CFI=0.963; SRMR=0.061 | | | | |
| AIC=6921.232; BIC=7041.448 | | | | |
| **Auditory Attention & Working Memory** | | | | |
| Intercept for cognitive performance | 0.588 | 0.049 | **<0.001** | (0.491, 0.684) |
| Slope for cognitive performance | 0.400 | 0.051 | **<0.001** | (0.300, 0.500) |
| *Effect of mHT allocation during KEEPS trial on slope for cognitive performance* |  |  |  |  |
| oCEE | -0.073 | 0.058 | 0.210 | (-0.187, 0.041) |
| tE2 | -0.046 | 0.057 | 0.419 | (-0.159, 0.066) |
| *Effect of mHT allocation during KEEPS trial on later cognitive function* |  |  |  |  |
| oCEE | 0.031 | 0.051 | 0.543 | (-0.069, 0.131) |
| tE2 | 0.067 | 0.044 | 0.132 | (-0.020, 0.154) |
| **Fit Indices** | | | | |
| CFI=0.957; SRMR=0.048 | | | | |
| AIC=5593.496; BIC=5709.418 | | | | |

**S1 Table** (continued)

| Variable | Estimate | S.E. | P-Value | 95% Confidence Intervals |
| --- | --- | --- | --- | --- |
| **Verbal Learning & Memory** | | | | |
| Intercept for cognitive performance | 0.396 | 0.063 | **<0.001** | (0.274, 0.519) |
| Slope for cognitive performance | 0.444 | 0.082 | **<0.001** | (0.284, 0.603) |
| *Effect of mHT allocation during KEEPS trial on slope for cognitive performance* |  |  |  |  |
| oCEE | -0.087 | 0.056 | 0.122 | (-0.196, 0.023) |
| tE2 | -0.070 | 0.055 | 0.205 | (-0.179, 0.038) |
| *Effect of mHT allocation during KEEPS trial on later cognitive function* |  |  |  |  |
| oCEE | -0.087 | 0.054 | 0.106 | (-0.192, 0.018) |
| tE2 | -0.070 | 0.056 | 0.210 | (-0.180, 0.040) |
| **Fit Indices** | | | | |
| CFI=0.978; SRMR=0.040 | | | | |
| AIC=8198.018; BIC=8313.940 | | | | |
| **Global Cognition (Modified Mini-Mental State Test)** | | | | |
| Intercept for cognitive performance | 0.867 | 0.320 | **0.007** | (0.239, 1.393) |
| Slope for cognitive performance | 0.254 | 0.509 | 0.618 | (-0.744, 1.091) |
| *Effect of mHT allocation during KEEPS trial on slope for cognitive performance* |  |  |  |  |
| oCEE | 0.226 | 0.176 | 0.200 | (-0.119, 0.516) |
| tE2 | 0.105 | 0.117 | 0.368 | (-0.124, 0.297) |
| *Effect of mHT allocation during KEEPS trial on later cognitive function* |  |  |  |  |
| oCEE | 0.032 | 0.091 | 0.722 | (-0.146, 0.183) |
| tE2 | -0.112 | 0.073 | 0.128 | (-0.256, 0.009) |
| **Fit Indices** | | | | |
| CFI=0.990; SRMR=0.039 | | | | |
| AIC=10361.520; BIC=10481.160 | | | | |

See Table 2 for abbreviations. The statistically significant findings are in bold font.
