# Supplementary material for: Long-term cognitive effects of menopausal hormone therapy: Findings from the KEEPS Continuation Study
Source: PLoS Med. 2024 Nov 21;21(11):e1004435. doi: 10.1371/journal.pmed.1004435 (PMC11581397; doi:10.1371/journal.pmed.1004435)
Supplement: Attachment — Submitted filename: PlosMed.Reviewer response--revision.docx [file pmed.1004435.s005.docx]

Thank you for positive comments. Below is a point-by-point response to the reviewer and editorial comments. NOTE: when we refer to a specific page, we are referencing the **Revised manuscript (marked-up copy)** (i.e., "Track Changes" version of the revised manuscript). For convenience we also submit a Revised manuscript (clean copy) that does not show changes.

**Academic Editor and Reviewer Comment:** Both Reviewer 1 and the Academic Editor note the importance of addressing the potential influence of attrition on the results.

*Reviewer #1: "It seems that the n=275 left for FU after 10 years were not different from the baseline cohort apart from baseline global cognition and SBP and that should perhaps be a little more highlighted in the paper, as this is very important. Could that have affected outcomes? Would those with poor cognition and drop-out be more likely not to want to/be able to return? This group had the highest opportunity to improve over time so that could be healthy survivor bias perhaps? Also could stroke after MHT (with the higher BP) have resulted in those at risk dropping out not showing a potential worsening over time?"*

*Academic Editor: " The academic editor ... felt that more needed to be done to address the issue that those who did not take part and who died had unknown cognitive decline. She wondered if the authors could do anything more to address this? She suggested that perhaps sensitivity analyses may be a useful approach, drawing on existing data from other studies where dropout has been shown to be associated with cognitive decline. She postulated that potentially it may be that, at one extreme end, the reasons for dropout/decline might actually reinforce the findings in this paper. But she was concerned that, in other possible scenarios, the dropout could possibly have skewed the results here. She also wondered whether the authors might be able to use death certification and recorded causes of death to help enlighten us on this issue."*

**Response:** This is such an interesting and important discussion point. Differences between those who return and do not return for visit a decade later can indeed influence findings. We expected the healthy survivor bias to be expressed as a result of the extended time without contact with participants. Surprisingly, as Reviewer 1 notes, the participants who returned were generally representative of the original cohort, except for differences in baseline global cognition and SBP. Although the p-values were not corrected for multiple comparisons, these differences, particularly in SBP, are in the direction of a healthy survivor bias, i.e., those with higher baseline SBP and worse baseline cognition may have been less likely to return due to stroke (as Reviewer 1 suggests) or other health factors related to cognitive decline.

Conversely, the influence of the survivor bias may have been reduced as function of the design of the KEEPS. The original KEEPS participants were selected for participation based on their low-CVD risk profile. For example, use of tobacco was very limited among participants and women with diabetes and elevated BMI were excluded. This high bar for health status at baseline may have mitigated the impact of the healthy survivor effects to some degree. Moreover, the women from the KEEPS are still relatively young. Mean age at follow-up was 67 (2). Based on 2020 census data, we would expect ~56 deaths to have occurred in a random group of 727 women between the ages of 55 and 64 over a ten-year period.^[1]^ We were informed of only 10 deaths. It is possible that there were many more deaths in the 105 individuals who we were not able to reach. Most likely the picture is likely distorted by both conditions: 1) underreported deaths in the lost-to-follow-up group and 2) the circumstance that women in the original KEEPS study were healthier than the general population.

Another factor worth considering as we attempt to understand the effect of attrition, is the COVID-19 pandemic. A number of KEEPS participants opted not to return based on concerns about COVID-19. Unlike other factors affecting availability for a research study such as general health, it is possible that COVID-19 concerns influenced attrition differently. Maybe the healthiest or those most vigilant of health stayed home? Similarly, some participants from the KEEPS were lost to follow-up not because of their circumstances, but because the original site PI moved or died. NOTE: We have added text in the limitation section of the discussion, elaborating on this point: “*Differences in baseline global cognition and SBP between participants and non-participants may reflect a healthy survivor bias, although some non-participation was due to factors not typically encountered in longitudinal follow-up studies, e.g., the COVID-19 pandemic, missing contact information because the PI moved to another institution or passed away*.”. These changes were made on **page 30** in the revised manuscript, as indicated in the Track Changes version.

Unfortunately, suggestions made by the editor and reviewer to investigate the effects of attrition are not feasible. Individual sites hold the participant names and identifiers. The individual site personnel would have to research causes of death for their participants. When study funding ended, personnel were re-assigned or let go. We no longer can ask them to commit time to the project. Likewise, because sites provided only aggregate data, we have no way of tracking down medical records, including stroke history for those who declined to participate or those who did not respond to contact attempts. Again, we would need to rely on the now disbanded study teams who hold the individual participant identifiers.

With regards to incident stroke, we can only comment on self-reported stroke occurring in the time between the KEEPS and KEEPS Continuation for those women who returned for the KEEPS Continuation. Only 5 women indicated a having had a stroke. Of those, 2 were randomized to mHT v. 3 women who had been assigned to placebo. OF NOTE: a manuscript providing MRI data is in preparation. The paper provides a clearer picture of detected cerebrovascular events compared to self-report. Findings presented it that manuscript indicate no difference in white matter hyperintensities or stroke between KEEPS randomization groups. Watch for paper by Faubion et al. "Long-term effects of short-term menopausal hormone therapy on white matter integrity.".

A. **Comments from Reviewer #1:**
1. **Reviewer Comment:** *"I may have missed ... but am wondering if the authors had data on VMS/sleep/mood and whether that modified these effects or whether that is part of another paper?"*

**Response:** We appreciate the reviewer's attention to detail and interest. Although VMS, sleep, and mood data are indeed relevant to the overall findings, we opted to conduct separate analyses. In this paper, we focused specifically on the cognitive outcomes from the KEEPS Continuation study. This approach aligns with the Specific Aims described in our grant proposal. We reported in the text that (**page 10** in the “Track Changes” version) “*Outcomes assessed included cognition and mood, and neuroimaging for Alzheimer's disease proteinopathies; analyses presented here are limited to the cognitive outcomes.*”.

2. **Reviewer Comment:** *"The 41% of participants who continued on MHT, how were they different? Could a GLM be done using none-past and ongoing user category?"*

**Response:** We apologize for the confusion, and revised the text in the Method section (see **page 15** of the revised “Track Changes” version of the manuscript. Text now reads as follows: “*Out of the 299 participants in KEEPS Continuation, 41 participants (overall, 13.71% of 299) continued with the mHT regimens used in the KEEPS trial or switched to another type of systemic mHT after the end of the study (oCEE, n=17, 41.5% of 41; tE2, n=15, 36.6% of 41; and placebo, n=9, 22% of 41)*. *Most of the KEEPS Continuation participants who went on to use systemic mHT or switched to another type of systemic mHT after the end of the study (n=40 out of 275; oCEE, 42.5%; tE2, 35%; placebo, 22.5%) also had cognitive data in the original KEEPS trial.*”. Only 40 out of 275 participants, who had cognitive data both at KEEPS and KEEPS Continuation, continued mHT or switched to another type of systemic mHT after KEEPS. Due to low number of mHT use (n= 40 out of 275; self-reported dose/frequency were heterogenous), the only sensitivity analysis that was feasible was to test the effect of removing the mHT users from the analysis to determine the effect on parameter estimates. Given the low number of mHT users after the trial, we are reticent to conduct any further analysis.

4. **Reviewer Comment:** *"Lastly I often find that using compound scores somehow reduces sensitivity of tests to change. Could the recall score be analysed separately rather than within the compound score?"*

**Response:** There are different types of composite measures based on methods used to combine the components. Composite measures require a strong foundation of reliability, validity and usefulness. We followed recommended best practices to develop composite measures (Forum, 2013): http://www.qualityforum.org/Publications/2013/04/Composite_Performance_Measure_Evaluation_Guidance.aspx.

We used factor scores obtained from rigorous multi-step latent variable modeling approaches described in our previous publications describing the KEEPS cognitive findings (Gleason et al., 2015). More specifically, we used structural equation modeling (SEM) techniques to reduce measurement error and increase the reliability and construct validity of the derived scores. The verbal learning and memory factor score used in the current paper included the California Verbal Learning Test Trials 1 to 3, and Long Delay recall; and also the New York University Paragraph Recall. The solution for the full battery of tests provided an excellent fit for both the global and individual factor scores.

It is true that a statistical disadvantage of composite scoring is that information from the individual items that make up the composite score is lost, and subsequent analyses of the composite score are less able to account for measurement error. However, we used advanced statistical techniques (SEM with latent variables) to mitigate such issues and increase the reliability of the composite. A future project might entail looking at how sensitive each of our derived factor scores and its indicators are in capturing change by looking at each component separately using, for example, approaches shown in Dowling et al. 2016 assessing change sensitivity in the ADAS-Cog battery (Dowling, 2016).

**Comment from Reviewer #2 (Statistical Review):**

1. **Reviewer Comment:** *"Abstract line 49-50: can it be clarified whether there were 2 or 3 randomized groups (it wasn't clear to me whether the two MHTs were distinct treatment arms or there was one MHT arm which could then choose between MHTs."*

**Response:** We have clarified in the abstract that "….participants were randomized into three groups...”. There were two distinct mHT treatment arms (oCEE and tE2) and one placebo arm. This information is now more clearly stated (**page 2**) in the “Track Changes” version of the revised manuscript.

2. **Reviewer Comment:** *"Abstract: it would be good to provide estimated associations and between-group differences (together with 95% CIs and p-values) in the results section."*

**Response:** Our initial response to this comment was to add the 95% CI and p-values for those estimates describing the influence of mHT randomization on slope of cognitive performance across the KEEPS and KEEPS Continuation visit into the abstract section, as requested by Reviewer #2. However, this resulted an error message in our resubmission. Specifically, “The abstract is too long. You cannot complete your submission until the following items are corrected.”

The following text was removed [*For verbal attention and executive function, the effect of mHT allocation on cognitive function slope across all visits was -0.012 for oCEE (95% CI: -0.090 to 0.066, p = 0.760) and -0.046 for tE2 (95% CI: -0.126 to 0.033, p = 0.254). For speeded language and mental flexibility, the effect of mHT allocation on slope of cognitive function across all visits was -0.021 for oCEE (95% CI: -0.094 to 0.052, p = 0.579) and -0.032 for tE2 (95% CI: -0.112 to 0.048, p = 0.437). For auditory attention and working memory, the mHT allocation effects on the slope of cognitive performance for all visits were 0.011 for oCEE (95% CI: -0.082 to 0.104, p = 0.817) and 0.052 for tE2 (95% CI: -0.031 to 0.134, p = 0.222). For verbal learning and memory, the effect of mHT allocation on the slope of cognitive scores across all visits was -0.084 for oCEE (95% CI: -0.181 to 0.012, p = 0.086) and -0.076 for tE2 (95% CI: -0.177 to 0.024, p = 0.138). For global cognition, the mHT allocation effects on the slope were 0.0001 for oCEE (95% CI: -0.118 to 0.017, p = 0.995) and -0.103 for tE2 (95% CI: -0.228 to 0.022, p = 0.106).*]. We will follow the editor’s instructions as to whether we should include this text. Of note, estimates for the intersection and slope, and the estimates for the slope during the KEEPS visits are provided in the Results section in **Table 2** (**pages 20-21** in the “Track Changes” version of the revised manuscript).

3. **Reviewer Comment:** *"Was this study registered? If so please provide the registration information."*

**Response:** While the KEEPS was a clinical trial the KEEPS Continuation was an observational study and did not meet criteria for registration with ClinicalTrials.gov. We have included the registration information for the original KEEPS study in the manuscript. This following text has been added to the "Methods" section (**page 11)** in the Track Changes version with an explanation: “The original KEEPS study’s clinical trial registration number is NCT00154180. The KEEPS Continuation was an observational study and did not meet the criteria for registration with ClinicalTrials.gov.”. We also provided the registration number of KEEPS in the “Abstract” section (**Page 2**) in the “Track Changes” version of the revised manuscript.

4. **Reviewer Comment:** *"Page 14, line 262: I did not follow the percentages other than 'overall'. E.g., why is 41.5% given for oCEE if 41 is the total number across all arms?"*

**Response:** We have reviewed and corrected the percentages related to oCEE in the "Results" section for clarity. Please also see our response to the first reviewer (comment 2).

5. **Reviewer Comment:** *"Statistical methods: as latent growth models would probably not be well known to readers, it might be helpful to have supplementary material that describes the model and also what assumptions are made."*

**Response:** We have included supplementary material (**S1 Statistical Methods; page 37-38** in the “Track Changes” version of the revised manuscript) describing the growth model with a distal outcome used in our primary analysis and corresponding standard assumptions.

6. **Reviewer Comment:** *"Results: I'm not sure if the LGMs used naturally have any visualization of results, but I would have found it interesting to see the mean trajectory over time in the different groups."*

**Response:** The goal of our analysis was on linking **person-level changes** during hormone therapy (a latent variable or “slope”) with cognitive performance 14 years after hormone therapy completion. Specific information on the effect of HT on cognitive outcome trajectories have been previously reported in our primary KEEPS trial paper on cognitive results (please see (Gleason et al., 2015)). Compared to the placebo, we did not find a differential effect of HT on cognitive measures across treatment groups. Therefore, mean trajectories did not differ.

**Comment from Reviewer #3:**

1. **Reviewer Comment:** *"The abstract can be made more concise and clearer with only* *necessary information especially the Methods and findings section."*

**Response:** We have edited the abstract with the goal of making it more concise. We note, however, that another reviewer asked for more information to be added in to the Abstract. Changes are denoted in the "Track Changes" version of the revised manuscript (**page 2-4)**.

2. **Reviewer Comment:** *"The reason for the hypothesis that the two treatment formulations would affect cognition differentially, can be explained stronger. The absence of such a difference could also be explained with possible reasons in the discussion with appropriate references."*

**Response:** We have strengthened the rationale for hypothesizing differential cognitive effects of the two mHT formulations in the "Introduction" and “Discussion” sections. Please see the text on **page 10 and pages 28-29** in the “Track Changes” version of the revised manuscript.

3. **Reviewer Comment:** *"The criteria used to classify women as low CVD risk and the rationale for excluding those with high CVD risk could be elaborated."*

**Response:** We have added text to explain how the KEEPS study defined low CVD risk. Specifically, participants were selected for enrollment in the KEEPS based on stringent criteria to ensure low CVD risk. The changes were made on **page 13** in the revised manuscript, as indicated in the “Track Changes” version.

4. **Reviewer Comment:** *"What can be the possible reasons for the difference in impact of MHT and delayed HT on cognition?"*

**Response:** Although beyond the scope of this paper, the question is immensely important. The timing of mHT initiation may be a critical factor in its impact on cognition, as suggested by the 'critical window' or 'timing hypothesis.' This hypothesis suggests that mHT is more likely to confer cognitive benefits when initiated during the early postmenopausal period, a time when the brain may be more responsive to estrogens. Delayed mHT, on the other hand, may be less effective or even harmful. Briefly, one theory put forward by Brinton et al. (Rettberg et al., 2014) described estrogen as a ‘master regulator’ in metabolic processes. In the context of metabolic health, estrogen promotes numerous neuroprotective actions. However, if metabolism is dysregulated, estrogens promote detrimental and dysregulated metabolic effects.

Furthermore, neurobiological changes that occur with aging, such as increased amyloid deposition, mitochondrial dysfunction, and reduced synaptic plasticity, might render the brain less capable of benefiting from mHT initiated later in life. Additionally, delayed mHT may be less effective in reversing or mitigating the progression of subclinical cerebrovascular disease, which is more likely to be established in older women. A number of papers have tested or discussed the hypothesis, presenting data in support for the theory (Henderson et al., 2005; Henderson & Brinton, 2010; Hodis et al., 2015; MacLennan et al., 2006; Resnick & Henderson, 2002; Sherwin, 2009; Whitmer et al., 2011). Still, it is far from a settled issue. Text, briefly touching on the evidence for and against the theory, is available in the “Discussion” section (**see page 28-29** in the “Track Changes” version of the revised manuscript).

Our data do not support the critical window hypothesis. The cognitive impact of mHT may be more complex than initially thought and could be influenced by other factors such as individual variability in response to estrogen, baseline cognitive function, or the presence of subclinical cerebrovascular disease.

5. **Reviewer Comment:** *"Were all the participants cognitively healthy (absence of MCI or dementia)? If not could the frequency of cognitive impairment also be compared among the groups?"*

**Response:** Thank you for this question. We looked for evidence of dementia or MCI and the numbers of women for whom this was possible were too small to conduct analyses. Nonetheless, we describe our observation for the Reviewer.

As noted, the women enrolled in the KEEPS held low cardiovascular risk at baseline with higher than average education levels (mean years of education ~16). In other words, they are at lower than average risk for the cognitive syndromes of MCI and dementia. The average 3MSE score for those individuals returning for a KEEPS Continuation visit was 97.26 (SD=3.32) out of a 100 total possible points. Although there was no adjudication of cognitive diagnoses, none of the women who returned for the KEEPS Continuation reported a diagnosis of dementia in their medical history. We did not ask about MCI. In their self-reported medical history 2 of the 299 women answered that they were 'uncertain' if they were diagnosed with dementia. One of those women answered "uncertain" for most of her medical history questions. The second obtained a score of 100/100 on the 3MSE. All others denied a history of dementia. Of course, we do not know if the women who did not return had a diagnosis of dementia.

To address the possibility of missed or mis-reported diagnoses of dementia or MCI, we looked at individual cognitive scores. The lowest 3MSE score was 81/100. Only 4 women scored at or lower than the clinical cut point of 85/100. None of those women reported a diagnosis of dementia; one women reported a history of bi-polar disorder. We looked closer at demographic data for these 4 women. Education levels were reported to be between 11 and 16 years of education. We examined their performance on a list learning test, the California Verbal Learning Test (CVLT) to gauge their overall memory performance. Delayed recall for the word list is very sensitive to early MCI and dementia. Importantly, the version of the CVLT we used in the KEEPS was modified from what is typically given in clinical setting. Women had only 3 exposures in the learning trials in our study as opposed to 5 trials in the clinic setting. Thus, our participants' score can be expected to be lower than what they might obtain had they been given two more opportunities to learn the list.

You can see in the Table below that all but one of the four women obtained a T-Score within 2 SD of the average (T-Score between 30 and 70). It is possible that the participant whose 3MSE score of 84/100 and whose delayed recall T-score was 25 is showing signs of MCI. Of note, this is the participant who report that she is diagnosed with bi-polar disorder.

|  |  |  |  | CVLT delayed recall scores | | |
| --- | --- | --- | --- | --- | --- | --- |
|  | 3MSE | Age | educ | Raw Score | Scaled Score | Age/education corrected T-Score |
| 1 | 81/100 | 67 | 16 | 10 | 8 | 40 |
| 2 | 84/100 | 70 | 12 | 5 | 3 | 25 |
| 3 | 85/100 | 65 | 11 | 9 | 7 | 37 |
| 4 | 85/100 | 60 | 13 | 7 | 5 | 30 |

Overall, of those who returned for visits, there are few participants whose data appear consistent with either MCI or dementia. Again, we cannot comment on those women who did not return for the KEEPS Continuation.

6. **Reviewer Comment:** *"Could the baseline difference in 3MSE scores have influenced the results?"*

**Response:** Indeed, this is what we found (see **Page 22** in the Track Changes version of the revised manuscript). LGM model results indicated that, "the strongest predictor of cognitive performance in KEEPS Continuation was cognitive performance in KEEPS trial both at baseline (intercept) and across time (slope)."

7. **Reviewer Comment:** *"The significance of performing the cross-sectional analysis on cognitive functioning can be explained better. Would a longitudinal study have made differences compared to the current methodology."*

**Response:** The purpose of the secondary cross-sectional analysis presented in the study was simply to show “current cognitive status” differences by initial treatment allocation after 14 years post mHT. We anticipated that readers would want to see how the groups compared cognitively at a mean age of 67. We agree with the reviewer that the cross-sectional analysis is not linked to what happened in terms of “cognitive change” while trial participants were on mHT. For this reason, we highlight the longitudinal analyses as the primary outcome of interest. Having additional data points post mHT completion would have provided more detailed information on post-treatment cognitive trajectory changes by group, but the KEEPS Continuation study was only funded to collect data at one time point.

8. **Reviewer Comment:** *"A separate analysis for the people who continued HT (depending on duration of HT) could have added clarity to the time-since-menopause theories."*

**Response:** We agree with the reviewer. This is an important consideration for the design of future studies. In the present study, however, the sample sizes are too small to make meaningful inferences (n=40 out of 275).

**REFERENCES**

Dowling, N. M., Bolt, D. M., & Deng, S. (2016). An approach for estimating item sensitivity to within-person change over time: An illustration using the Alzheimer’s Disease Assessment Scale–Cognitive subscale (ADAS-Cog). *Psychological Assessment*, *28*(12), 1576-1585. <https://doi.org/https://doi.org/10.1037/pas0000285>

Forum, N. Q. (2013). Composite Performance Measure Evaluation Guidance. <https://www.qualityforum.org/publications/2013/04/composite_performance_measure_evaluation_guidance.aspx>

Gleason, C. E., Dowling, N. M., Wharton, W., Manson, J. E., Miller, V. M., Atwood, C. S., Brinton, E. A., Cedars, M. I., Lobo, R. A., Merriam, G. R., Neal-Perry, G., Santoro, N. F., Taylor, H. S., Black, D. M., Budoff, M. J., Hodis, H. N., Naftolin, F., Harman, S. M., & Asthana, S. (2015). Effects of Hormone Therapy on Cognition and Mood in Recently Postmenopausal Women: Findings from the Randomized, Controlled KEEPS-Cognitive and Affective Study. *PLoS Med*, *12*(6), e1001833; discussion e1001833. <https://doi.org/10.1371/journal.pmed.1001833>

Henderson, V. W., Benke, K. S., Green, R. C., Cupples, L. A., & Farrer, L. A. (2005). Postmenopausal hormone therapy and Alzheimer's disease risk: interaction with age. *J Neurol Neurosurg Psychiatry*, *76*(1), 103-105. <https://doi.org/10.1136/jnnp.2003.024927>

Henderson, V. W., & Brinton, R. D. (2010). Menopause and mitochondria: windows into estrogen effects on Alzheimer's disease risk and therapy. *Prog Brain Res*, *182*, 77-96. <https://doi.org/10.1016/s0079-6123(10)82003-5>

Hodis, H. N., Mack, W. J., Shoupe, D., Azen, S. P., Stanczyk, F. Z., Hwang-Levine, J., Budoff, M. J., & Henderson, V. W. (2015). Methods and baseline cardiovascular data from the Early versus Late Intervention Trial with Estradiol testing the menopausal hormone timing hypothesis. *Menopause*, *22*(4), 391-401. <https://doi.org/10.1097/gme.0000000000000343>

MacLennan, A. H., Henderson, V. W., Paine, B. J., Mathias, J., Ramsay, E. N., Ryan, P., Stocks, N. P., & Taylor, A. W. (2006). Hormone therapy, timing of initiation, and cognition in women aged older than 60 years: the REMEMBER pilot study. *Menopause*, *13*(1), 28-36. <https://doi.org/10.1097/01.gme.0000191204.38664.61>

Resnick, S. M., & Henderson, V. W. (2002). Hormone therapy and risk of Alzheimer disease: a critical time.[see comment][comment]. *JAMA*, *288*(17), 2170-2172.

Rettberg, J. R., Yao, J., & Brinton, R. D. (2014). Estrogen: a master regulator of bioenergetic systems in the brain and body. *Front Neuroendocrinol*, *35*(1), 8-30. <https://doi.org/10.1016/j.yfrne.2013.08.001>

Sherwin, B. B. (2009). Estrogen therapy: is time of initiation critical for neuroprotection? *Nat Rev Endocrinol*, *5*(11), 620-627. <https://doi.org/10.1038/nrendo.2009.193>

Whitmer, R. A., Quesenberry, C. P., Zhou, J., & Yaffe, K. (2011). Timing of hormone therapy and dementia: the critical window theory revisited. *Ann Neurol*, *69*(1), 163-169. <https://doi.org/10.1002/ana.22239>

1. Source: https://www.statista.com/statistics/241572/death-rate-by-age-and-sex-in-the-us/

   Annual number of deaths per 100,000 of population is 773.1 for women age 55-64.
