# Supplementary material for: Long-term cognitive effects of menopausal hormone therapy: Findings from the KEEPS Continuation Study
Source: PLoS Med. 2024 Nov 21;21(11):e1004435. doi: 10.1371/journal.pmed.1004435 (PMC11581397; doi:10.1371/journal.pmed.1004435)
Supplement: Attachment — Submitted filename: Response to editorial comments.docx [file pmed.1004435.s006.docx]

Thank you for positive comments. Below is a point-by-point response to the reviewer and editorial comments.

**Comments from the academic Editor**
**Comment 1:** *The academic editor looked through your rebuttal letter and thought that there was a lot of valuable information that could be incorporated into the main text of the manuscript for the benefit of the readers. For example, she suggests paraphrasing what you’d written in response to the request for information about attrition analyses to the paper’s existing discussion of the study’s limitations, i.e. to explain why this wasn’t feasible, etc.* 
**Response:** Thank you for your comments. This part in response letter has been summarized and added into the revised manuscript as a part of the limitation section.

**Comment 2:** *She also said the following: ‘They need to have a summary that reflects not only that harm isn't done but that cognition is not improved as there is still medication being recommended in some places to prevent cognitive decline - the findings suggest no effect so this needs to be considered in both directions of earlier proposed impact.’ Our suggestion is that the authors revise the last couple of lines of the Abstract to reflect this, as well as add this point to the Discussion and/or Conclusion.*

**Response:** Thank you for your comments. We revised the abstract and the Conclusion part accordingly.

**Comments from the Editors**
**Comment 1:**    *Data availability: Could we ask if you could provide different contact details for data access (i.e. not for a single author) please? When possible, we recommend authors deposit restricted data to a repository that allows for controlled data access. If this is not possible, directing data requests to a non-author institutional point of contact, such as a data access or ethics committee, helps guarantee long term stability and availability of data. Providing interested researchers with a durable point of contact ensures data will be accessible even if an author changes email addresses, institutions, or becomes unavailable to answer requests. Further information is available* [*here*](https://urldefense.com/v3/__https:/journals.plos.org/plosmedicine/s/data-availability*loc-faqs-for-data-policy__;Iw!!Mak6IKo!Onj1t-ACgY2H_WYdSVS2vM1E5kFdalCfGnAop4u0rpB33_EoOw_9vtgJ9dWuYMspicOMlQ2u_UtFBj8Ph4YV$)*:*

**Response:** We used Medidata as the data repository, and the Mayo Clinic Quantitative Health Sciences statistical team has full access to it. The team can provide the data upon request, but only after approval from one of the PIs of the project, Dr. Kejal Kantarci (kantarci.kejal@mayo.edu).

We have added the following text to the revised manuscript:
*"For data access, please contact Dr. Kejal Kantarci (kantarci.kejal@mayo.edu), who will serve as the point of contact. The data is stored in the Medidata repository, and any requests for access must be approved by Dr. Kantarci before being forwarded to the Mayo Clinic Quantitative Health Sciences statistical team, which manages access to the repository."*

Since Dr. Kantarci is currently away attending a conference, we may provide a non-author contact after her return on 10/23/24, if feasible.

-      Please address the following that was requested at major revision based on the study’s design:
OBSERVATIONAL STUDIES

**Comment 2:**     *Abstract: Please include the study design, population and setting, number of participants, years during which the study took place (enrollment and follow up), length of follow up, and main outcome measures.*
**Response:** Thank you for your comments. To add the requested information regarding the study design, and years during the study took place, we revised the abstract. Due to word limitation, the hypothesis section has been shortened.

**Comment 3:**     *Please ensure that the study is reported according to the STROBE (or appropriate STROBE extension) guideline (available* [*here*](https://urldefense.com/v3/__https:/www.equator-network.org/reporting-guidelines/strobe__;!!Mak6IKo!Onj1t-ACgY2H_WYdSVS2vM1E5kFdalCfGnAop4u0rpB33_EoOw_9vtgJ9dWuYMspicOMlQ2u_UtFBiz5hK9Y$%20))*) and include the completed STROBE (or STROBE extension) checklist as Supporting Information. Please add the following statement, or similar, to the Methods: "This study is reported as per the Strengthening the Reporting of Observational Studies in Epidemiology (STROBE) guideline (S1 Checklist)." When completing the checklist, please use section and paragraph numbers, rather than page numbers.*

**Response:** STROBE guideline has been added into the supplementary materials.

**Comment 4:**  *For all observational studies, in the manuscript text, please indicate: (1) the specific hypotheses you intended to test, (2) the analytical methods by which you planned to test them, (3) the analyses you actually performed, and (4) when reported analyses differ from those that were planned, transparent explanations for differences that affect the reliability of the study's results. If a reported analysis was performed based on an interesting but unanticipated pattern in the data, please be clear that the analysis was data driven.*
**Response:** Thank you for the comments.

(1) The main hypothesis we intended to test was presented in the text in the introduction section.

(2) and (3): The Latent Growth Model (LGM) with a distal outcome approach was selected over the originally planned linear mixed effect (LME) for several reasons all related to the phenomenon that participants were re-evaluated after 10+ years with no monitoring. Primary among these were, 1) LGMs are better suited for fitting models with a distal outcome [1]; and 2) LGM allows us to observe non-linear changes in cognition, and 3) LGM approach permits us to model change as a random effect [2].

(4) The results did not confirm our hypothesis. We have discussed this in our Discussion.

*For factors that could have affected our results such as the* continued use of systemic mHT or switching to another type of systemic mHT or starting to use a systemic mHT (from placebo group) after the end of KEEPS, we conducted sensitivity analyses (results reported in S3 Table.

To examine potential differences between KEEPS Continuation participants and the original KEEPS, we compared baseline characteristics between women who returned for the KEEPS Continuation and the non-participants enrolled in the original KEEPS for whom we had KEEPS baseline cognitive data. We found that KEEPS baseline characteristics of women returning for the KEEPS Continuation were similar to those not returning with the exception of blood pressure readings and baseline 3MSE scores.

**Comment 5:**  *Please state in the Methods section whether the study had a prospective protocol or analysis plan. If a prospective analysis plan (from your funding proposal, IRB or other ethics committee submission, study protocol, or other planning document written before analyzing the data) was used in designing the study, please include the relevant document(s) with your revised manuscript as a Supporting Information file to be published alongside your study and cite it in the Methods section. A legend for this file should be included at the end of your manuscript. If no such document exists, please make sure that the Methods section transparently describes when analyses were planned, and when/why any data-driven changes to analyses took place. Changes in the analysis, including those made in response to peer review comments, should be identified as such in the Methods section of the paper, with rationale.*
**Response:** In our resubmission, we have included our protocol which includes a description of planned analyses. The specific analysis plan relevant to this manuscript can be found on page 20 of the protocol titled *"KEEPS_Continuation_Protocol_2017Nov08_for_centers,"* under the section " **Statistical Analysis for Objective 2.**"

We note that our original analysis plan included a linear mixed effect modeling (LME) approach. We used a latent growth model (LGM) analysis instead. Although not identical, both approaches are used to examine growth/change over time. In contrast to LME use of regression models, the LGM model relies on a structural equation modeling framework using confirmatory factor analysis (CFA) models with a mean factor structure and specific constraints to estimate “growth” or change across time. LGM provides an estimate of individual-specific deviation from the average growth curve and random effects are specified as latent variables in a CFA, instead of randomly varying coefficients in the traditional LME model. Both conceptualizations have been shown to be mathematically equivalent [3].

We have added the text explaining this alteration of the planned analyses to the Supplementary document titled, "S2 Statistical Methods":

“The LGM approach was selected over LME for several reasons all related to the phenomenon that participants were re-evaluated after 10+ years with no monitoring. Primary among these were, 1) LGM are better suited for fitting models with a distal outcome [1]; and 2) LGM allows us to observe non-linear changes in cognition, and 3) LGM approach permitted us to model change as a random effect [2].”

**Comment 6:**     *Can you also clarify whether the authors had contact with the patients or not, please? Forgive me for my confusion – it seems like there might have been no contact in some instances but at the same time someone must have contacted the participants for the purpose of being involved in this study? Can you also verify that appropriate consent processes were in place and/or elaborate (both in your rebuttal and in the manuscript) on this please?*
**Response:** Apologies for creating confusion. Site PIs and their research staff had contact with the participants enrolled at their site. In most instances the site PIs was the same person who led data collection for the original KEEPS. Each site obtained IRB approval to re-contact the participants enrolled at their site. Participants signed site-specific consent forms reviewing all study procedures and the sharing of data with the Mayo Clinic and the University of Wisconsin, Madison, allowing all sites' data to be collated for analyses. Dr. Kantarci served as site PI for the Mayo Clinic in place of Dr. Miller, the original KEEPS Mayo Clinic site PI. Outside of this subset of participants, the study MPIs (Dr. Gleason and Dr. Kantarci) did not have names or contact details of participants.

**Comment 7:**     *We note your request for clarification as to whether to add further results to the Abstract (for which you received an error from the system). Thank you for your efforts here. As there is a limit to the word count associated with Abstracts, this will have to be kept off the Abstract.*
**Response:** Thank you for the response. We kept this part off the abstract.

**Comment 8:** *In the Abstract, last line of the Methods and Findings section, please expand on the abbreviation ‘APOEe4’.*
**Response:** Thank you for the comment. We have expanded on the abbreviation.

**Comment 9:** *In the Author Summary, please expand on the first use of the abbreviate ‘HT’.*
**Response:** Thank you for the comment. We revised “HT” as “mHT” which was expanded on the first use.

**Comment 10:** *In the Author Summary, please also address this point initially raised at major revision: ‘Please revise your ‘What do these findings mean?’ section to comply with the following PLOS guidance: “Authors should reflect on the new knowledge generated by the research and the implications for practice, research, policy, or public health. Authors should also consider how the interpretation of the study’s findings may be affected by the study limitations. In the final bullet point of ‘What Do These Findings Mean?’, please describe the main limitations of the study in non-technical language.” ’*

**Response:** Thank you for the comment. We have revised this section and explicitly explain the new knowledge, implications for practice and research, and study limitations.

**Comment 11:**       *Please cite the reference numbers in square brackets. Citations should precede punctuation.*
**Response:** We have updated citations style. References numbers are in square brackets.

**Comment 12:**       *Please submit a clean version of the paper as the main article file. A version with changes marked must also be uploaded as a marked-up manuscript file.*

**Response:** We uploaded both marked-up and a clean version.

**Comment 13:**       *we ask that you provide a short, non-technical Author Summary of your research to make findings accessible to a wide audience that includes both scientists and non-scientists. The Author Summary should immediately follow the Abstract in your revised manuscript. This text is subject to editorial change and should be distinct from the scientific abstract.*

**Response:** This was provided previously. It has been revised.

**Comment 14:**       *We ask every co-author listed on the manuscript to fill in a contributing author statement. If any of the co-authors have not filled in the statement, we will remind them to do so when the paper is revised. If all statements are not completed in a timely fashion this could hold up the re-review process. Should there be a problem getting one of your co-authors to fill in a statement we will be in contact. YOU MUST NOT ADD OR REMOVE AUTHORS UNLESS YOU HAVE ALERTED THE EDITOR HANDLING THE MANUSCRIPT TO THE CHANGE AND THEY SPECIFICALLY HAVE AGREED TO IT.*

**Response:** We did not add or remove authors.

**Comment 15:**       *Please review your reference list to ensure that it is complete and correct. If you have cited papers that have been retracted, please include the rationale for doing so in the manuscript text or remove these references and replace them with relevant current references. Any changes to the reference list should be mentioned in the rebuttal letter that accompanies your revised manuscript.*

**Response:** It has been reviewed. The reference is complete and correct. We did not cite papers that have been retracted. We added new references in the supplementary document to respond comment 5.

**Comment 15:**       *Please note, when your manuscript is accepted, an uncorrected proof of your manuscript will be published online ahead of the final version, unless you've already opted out via the online submission form. If, for any reason, you do not want an earlier version of your manuscript published online or are unsure if you have already indicated as such, please let the journal staff know immediately at plosmedicine@plos.org.*
**Response:** We agree to have the uncorrected proof published online ahead of the final version.

**References**

1.	McCormick EM, Curran PJ, Hancock GR. Latent growth factors as predictors of distal outcomes. Psychol Methods. 2024.

2.	McNeish D, Matta T. Differentiating between mixed-effects and latent-curve approaches to growth modeling. Behavior Research Methods. 2018;50(4):1398-414.

3.	Curran PJ. Have Multilevel Models Been Structural Equation Models All Along? Multivariate Behav Res. 2003;38(4):529-69.
